# Comprehensive analysis of putative dihydroflavonol 4-reductase gene family in tea plant

**DOI:** 10.1371/journal.pone.0227225

**Published:** 2019-12-26

**Authors:** Xin Mei, Caibi Zhou, Wenting Zhang, Dylan O’Neill Rothenberg, Shihua Wan, Lingyun Zhang

**Affiliations:** 1 South China Botanical Garden, Chinese Academy of Sciences, Guangzhou, Guangdong, China; 2 College of Horticulture Science, South China Agricultural University, Guangzhou, Guangdong, China; 3 Department of Tea Science, Qiannan Normal University for Nationalities, Duyun, Guizhou, China; Zhejiang University, CHINA

## Abstract

One identified dihydroflavonol 4-reductases (DFR) encoding gene (named as *CsDFRa* herein) and five putative *DFRs* (named as *CsDFRb1*, *CsDFRb2*, *CsDFRb3*, *CsDFRc* and *CsDFRd*) in tea (*Camellia sinensis*) have been widely discussed in recent papers concerning multi-omics data. However, except for *CsDFRa*, their function and biochemical characteristics are not clear. This study aims to compare all putative *CsDFRs* and preliminarily evaluate their function. We investigated the sequences of genes (coding and promoter regions) and predicted structures of proteins encoded, and determined the activities of heterologously expressed CsDFRs under various conditions. The results showed that the sequences of five putative *CsDFRs* were quite different from *CsDFRa*, and had lower expression levels as well. The five putative CsDFRs could not catalyze three dihydroflavonol substrates. The functional CsDFRa had the strongest affinity with dihydroquercetin, and performed best at pH around 7 and 35°C but was not stable at lower pHs or higher temperatures. Single amino acid mutation at position 141 modified the preference of CsDFRa for dihydroquercetin and dihydromyricetin, and also weakened its stability. These data suggest that only CsDFRa works in the pathway for generating anthocyanidins and catechins. This study provides new insights into the function of *CsDFRs* and may assist to develop new strategies to manipulate the composition of tea flavonoids in the future.

## Introduction

Flavonoids are characteristic secondary metabolites in tea [*Camellia sinensis* (L.) O. Kuntze]. They, including the well-known catechins, not only play an important role in tea plant physiology, but also greatly contribute to the flavour and health function of tea products. In recent years, another category of flavonoids, anthocyanidins, and their glycosides, anthocyanins, have attracted researchers’ great interest for their high content in purple tea leaf [1]. Besides their great ornamental value, anthocyanins are important secondary metabolites for mitigating naturally occurring stresses to the plant [2–5]. In human body, the antioxidant property of anthocyanins promotes the health function of tea consumption, contributing to prevention against cardiovascular and inflammatory diseases [6]. Moreover, anthocyanidins can be further converted into catechins and proanthocyanidins. Therefore, in order to improve tea quality and function, it is important to optimize the composition and ratio of anthocyanidins. Thus, the molecular mechanisms for anthocyanin accumulation in purple-leaf tea varieties have been an active topic of research in recent years [7–9].

The flavonoid biosynthesis pathway (ko00941) on the KEGG website depicts that the main flavonoids, such as anthocyanidins, anthocyanins, catechins, epicatechins, and proanthocyanidins (condensed tannins), are all synthesized from three dihydroflavonols (DHFs), i.e., dihydrokaempferol (DHK), dihydroquercetin (DHQ) and dihydromyricetin (DHM). Only one enzyme, dihydroflavonol 4-reductase (DFR; EC1.1.1.219), catalyzes the reduction of these three DHFs to corresponding leucoanthocyanidins which are leucopelargonidin, leucocyanidin and leucodelphinidin, respectively. Leucoanthocyanidins are subsequently converted into their respective anthocyanidins and other flavonoids. In light of its substrate specificity, DFR, in a sense, controls the flux into three biosynthetic branches leading to diverse anthocyanidins and catechins [10]. DFRs from diverse species exhibit different substrate preference. So far, at least eight anthocyanins have been identified from purple tea, and the main aglycones are cyanidin and delphinidin [11–12].

The same pathway in horticultural plants has been illustrated in several published papers [13–15], where transcriptomic profiles were depicted and enlightened researchers to carry out further experiments. From these publications, several *CsDFR* members were revealed. DFR is usually encoded by a gene family in plant species [16]. In spite of several putative *CsDFR* isoforms discovered in RNA-seq data and discussed on assuming that they had performed DFR function, there has been only one *CsDFR* (named *CsDFRa* herein) reported so far [17–19]. From previous publication and homologous comparison, we have found another five genes as putative DFR genes in tea plant (see the Results section for their GenBank Accession No.). However, their sequences greatly differ from *CsDFRa*, and whether they have the ability to form leucoanthocyanidins has not been clear. This might cause some misjudgment in the analysis of transcriptome data.

Besides, amino acid residues 134 and 145 (*Gerbera* DFR numbering) play important roles in the substrate specificity [20]. The mutation at site 145 (Glu to Leu) resulted in white flowers, and this site is generally conserved in various plants, including tea. On the other hand, a different mutation at site 134 changed the preference of DFR and modified its flux-controlling role. But there is no consistent conclusion about the effect of this mutation in different plants [21], nor clear evidence for what would happen when CsDFRa was mutated at the corresponding site, the 141th residue.

In this study, we aimed to investigate all putative *CsDFRs* to preliminarily understand each member’s function, and investigate the effect on substrate specificity when a single amino acid was mutated at position 141 of CsDFRa. Through comparing the gene and protein structures of putative *CsDFRs*, analyzing their promoter sequences and expression profile, and determining the kinetics of CsDFRa and its two mutants, we concluded that only CsDFRa was able to reduce DHFs, or more precisely, DHQ and DHM, while other five putative CsDFRs did not generate anthocyanidins and should not be considered in transcriptome analysis. Additionally, N141 mutation was found to change CsDFRa’s substrate specificity.

## Materials and methods

### Bioinformatic analysis

The bioinformatic analysis of *CsDFRs* was implemented in accordance to previous publication [22]. Six *CsDFR* candidates were retrieved from genome of *Camellia sinensis* var. *sinensis* cv. Shuchazao (SCZ; Acc. No.: PRJNA510226). After analysing the position of conserved motifs, the structures of mRNAs and promoters were drawn by using Gene Structure Display Server (GSDS: http://gsds.cbi.pku.edu.cn/) [23].

ClustalW in MEGA 7.0 was used to align multiple sequences of CsDFR genes and proteins, and then a phylogenetic tree was made with NJ method and labelled by using FigTree.

Multiple Em for Motif Elicitation (MEME: http://meme-suite.org/tools/meme) [24] was employed to identify the conserved motifs of CsDFR proteins, and parameters were set as: Site distribution = Any Number of Repetitions (anr); The number of motifs to find = 20; The width of motif = 6–200 residues [22]. Motifs were then annotated by HMMER website (HMMER: https://www.ebi.ac.uk/Tools/hmmer/search/hmmscan). Transmembrane helices in proteins were predicted by TMHMM (http://www.cbs.dtu.dk/services/TMHMM/). Prediction of the subcellular location of eukaryotic proteins were run on TargetP (http://www.cbs.dtu.dk/services/TargetP/) [25].

Promoters of CsDFR genes were analysed as follows: Upstream sequence (2000 bp) of each coding sequence was retrieved from SCZ genome data (except for CsDFRb1, where only 1674 bp was detected). PlantCARE (http://bioinformatics.psb.ugent.be/webtools/plantcare/html/) was employed to predict cis-elements in these promoter regions. A BED (Browser Extensible Data) file (S2 File) containing some cis-elements’ positions were uploaded to GSDS (as described above) and drew a distribution picture.

The expression data extracted from Tea Plant Information Archive (TPIA; http://tpia.teaplant.org) were scaled by logarithm before being illustrated in heatmaps as described before [26].

The molecular models of CsDFRa were built by using EasyModeller [27] with the following chains from PDB (Protein Data Bank): 2C29_D (for DHQ) / 2IOD_D (for DHM), 2RH8_A, 2P4H_X, 4QTZ_A, 4QUK_A and 4R1S_A. The molecular docking of DFR with substrates DHQ or DHM was performed by AutoDock version 4.2 [28].

### Prokaryotic expression

First of all, expression plasmids were constructed and proteins were purified. RNA was extracted from tea leaves of Baitang purple tea (BTP) variety [15] grown in the Teaching and Research Station of South China Agricultural University (Guangzhou, China), by using an RNA extraction kit (Cat. # ZH0109, Huayueyang Biotechnology Co., LTD., Beijing, China). Total cDNA was then synthesized by using a PrimeScript^TM^ RT reagent Kit (Cat. # RR047A, TaKaRa). All primers for cloning putative *CsDFRs* have been listed in S2 File. First, primers in UTR of *CsDFRs* were designed according to the tea transcriptome database, and employed to isolate and amplify target genes by a high fidelity PrimeSTAR^®^ Max DNA Polymerase (Cat. # R045A, TaKaRa). The PCR products were isolated by agarose gel electrophoresis and purified with a Biospin Gel Extraction Kit (Cat. # BSC0M1, Bioer Technology Co. Ltd.), and added with dATP at 3’ termini by a normal Taq enzyme (Cat. # 12007, Microanalysis Inc.). After being ligated with pMD-18T vector (Cat. # 6011, TaKaRa) and transformed into competent cells (Shanghai Weidi Biotechnology Co., Ltd.) of *E*. *coli* strain DH5α, the newly constructed plasmids were extracted and purified by using BioSpin Plasmid DNA Extraction Kit (Cat. # BSC01M1, Bioer Technology Co., Ltd.), and sent for sequencing to get the open reading frame (ORF) sequences. Then, new primers were designed for introducing the ORF of *CsDFRs* into pET-32a (Novagen, Madison, WI, USA) which had been linearized by high fidelity PCR first. The recombinants were replicated in DH5α and then extracted and transformed into *E*. *coli* strain Rosetta (DE3). Induced by 0.1 mM of IPTG (Isopropyl-beta-D-thiogalactopyranoside. Biosharp Life Sciences Co., Ltd.) at 18°C for 16 h, CsDFR proteins were expressed and then extracted by ultrasonication (Ø 3 mm, 20% power, working and interval time 1 s / 2 s, total 20 min) with 1 mg/mL of lysozyme and an EDTA-free Protease Inhibitor Cocktail (Roche, Basel, Switzerland) added. After centrifugation, the supernatant was purified through Ni Sepharose 4B (45–165 μm bead diameter) columns (Cat. # MR035, Beijing Dingguo Biotechnology Co., Ltd) and PD-10 desalting columns (Cat. # 17-0851-01, GE Healthcare) successively. Concentrations of purified proteins were determined by Pierce^TM^ BCA Protein Assay Kit (Prod. # 23227, Thermo Scientific, Rockford, USA). Other relating details could be found in each kit’s instruction.

### Enzyme activity assay

The catalytic function of heterologously expressed CsDFRs was briefly identified by observing colour. DHFs (DHK, DHQ, DHM) were dissolved in methanol to 80 mM [16]. Total volume of 200 μL reaction solution contains CPBS (0.1 M citric-acid / 0.2 M disodium hydrogen phosphate buffer solution, pH 7.2), 30 μg CsDFR enzyme purified from Ni Sepharose, 1 mM DHK or 0.4 mM DHQ/DHM, and 2 mM NADPH·Na_4_. The reaction was carried out at 37°C for 1 h, and stopped and extracted by adding 200 μL of n-butanol:37% HCl (95:5, v/v). The mixture was incubated at 95°C for 15 min to convert leucoanthocyanidins, the colourless products of DFR, into coloured anthocyanidins [19, 29].

Kinetics of putative CsDFRs was investigated under atmospheric conditions. One milliliter of reaction mixture contained CPBS (pH 7.2), 0.01~0.4 mM substrate, 5 μg CsDFR enzyme, and 0.24 mM NADPH·Na_4_. The oxidation of NADPH was determined in a quartz cuvette (5 mm) at 335 nm at 25°C for 30 min and first five minutes with good linearity were taken into calculation. The enzyme activity was calculated by using the extinction coefficient of NADPH, 6.22 mM^-1^ cm^-1^ [18]. Specific activity as the units per microgram enzyme.

Optimum pH and temperature were examined as above under atmospheric conditions except for the concentrations of substrates were 0.1 mM (DHK was 0.2 mM). A higher concentration may exceed the detection limit. During the pH test, self-degradation of NADPH happened in acidic environment, especially at pH 4.0. Thus, the self-degradation rate was subtracted.

In the pH stability experiment, 5 μg enzyme was pipetted into 100 μL buffer with different pHs (4.0, 6.0, 7.0, 8.0) and kept on ice for 30 min. Then, the solution was adjusted to 1 mL reaction system with pH 7.2 as described in kinetics investigation. In the thermal stability test, CPBS, whose pH changed little at high temperatures, was pre-heated at various temperatures (25, 35 and 45°C). The reaction mixture was prepared and detected at 330 nm. Then it was put into water bath again and detected five minutes later. The original enzyme stored in −40°C refrigerator and thawed on ice was used as the control.

All the assays were repeated three times. One-way ANOVA and Tukey's test in SPSS 11.5 were used to check the significant difference (*p* < 0.05).

### Subcellular localization

Subcellular localization was detected as described before [26]. Briefly, *CsDFRa* and *CsDFRaΔ87* (lacking 87 nucleotides at N-terminal) were ligated into pSAT6-EYFP-N1 vector, respectively. Primers were listed in S2 File. The methods for constructing and purifying plasmids were the same as described in Section 4.2. Each 100 μL of recombinant plasmid was concentrated in a centrifugal concentrator at 45°C and 1300 rpm and vacuumed for 2 h to 10 μL. They were then transformed into *Arabidopsis thaliana* protoplasts. The YFP fluorescence was observed at 579 nm under a confocal microscope.

## Results

### Gene sequence analysis of putative *CsDFRs*

After carefully searching and comparing, one identified and five putative *CsDFR* genes, possibly responsible for reduction of DHFs, were picked up from transcriptome data on the NCBI website and tea genome, including var. *sinensis* (CSS) and *assamica* (CSA). The results were mapped to CSS genome data to obtain the final sequences of coding regions and promoters. For convenience, they were herein temporarily designated as *CsDFRa*, *CsDFRb1*, *CsDFRb2*, *CsDFRb3*, *CsDFRc*, *CsDFRd*, based on their similarity and the names already existed in database. *CsDFRa* has been confirmed and reported in previous researches [17–19]. Three *CsDFRbs* are highly similar to each other and were predicted as *DFRs* in the database. The open reading frames of *CsDFRb2* and *CsDFRb3* share the highest similarity of 85.5% for their gene sequences. The conserved regions of *CsDFRbs* are 68.4%~70.1% similar to that of *CsDFRa* detected by using the discontiguous megablast program (more dissimilar) but no significant similarity was found by using the megablast program (highly similar) in Basic Local Alignment Search Tool (BLAST) (Table 1). *CsDFRc* and *CsDFRd* have little similarity to *CsDFRa*, and according to the homologous comparison to other plants, they are more likely to be cinnamyl-alcohol dehydrogenase and short-chain dehydrogenases/reductases, respectively. However, considering their similarity to *DFRs* in some plants and the annotation as *DFRs* in the tea database, we investigated them together with *CsDFRa* and *CsDFRbs* in the following analysis.

**Table 1:** Accession information for putative *CsDFRs* and predicted protein parameters.

Name (Acc. No.)	ID (locus) in SCZ genome (PRJNA510226)	Per. Ident of genes	Per. Ident of proteins	Protein		
				Length (a.a.)	Mol. Wt. (kDa)	pI
CsDFRa (AB018685.1)	TEA032730 (Scaffold1618:1281798:1287743:+)	100%	100%	347	38.7	6.02
CsDFRb1 (XM_028251603.1)	TEA023829 (Scaffold349:1768973:1773282:-)	68.4%	59.6%	344	38.5	5.82
CsDFRb2 (XM_028243762.1)	TEA024758 (Scaffold7032:133075:135417:-)	67.8%	58.9%	344	38.5	5.70
CsDFRb3 (XM_028243764.1)	TEA024762 (Scaffold7032:101142:108302:-)	70.1%	56.9%	339	37.5	5.75
CsDFRc (XM_028268820.1)	TEA010588 (Scaffold984:1305738:1314349:-) TEA022775 (Scaffold1059:578867:603519:+)	-	43.2%	357	39.1	6.35
CsDFRd (XM_028230958.1)	TEA003656 (Scaffold3763:214114:217562:-)	-	25.5%	339	36.8	7.04

The accession numbers (Acc. No.) of six *CsDFR* genes in tea genome were provided in Table 1, where also listed are their annotation IDs and location coordinates in the genome data of *Camellia sinensis* var. *sinensis* cv. Shuchazao (SCZ; Acc. No.: PRJNA510226) [30]. *CsDFRb2* and *CsDFRb3* are located in the same scaffold (Scaffold7032), and *CsDFRc* has two loci (Scaffold984 and Scaffold1059).

Before further analysis, these genes were all cloned from tea leaves and their nucleotide sequences were identified. By using the primers designed in untranslated regions (UTR), two types of *CsDFRa* with different length were isolated in our tea material (S1 File). One type is similar to the *CsDFR* detected in the genome of var. *assamica* (not spliced completely and located in the scaffolds of Sc0001530 and Sc0001101), and has been published in previous papers (exactly the same as AB018685.1 in the var. Yabukita) [17–19]. The other type is a new discovery, where 21 bases, i.e., 7 amino acid residuals (PVNGNKV) are missing at C-terminus and the same occasion was detected in the genome of var. *sinensis* (*CsDFRa* Acc. No.: XM_028203817.1). We sent 10 colonies for sequencing and the longer type occupied 1/3. Since there was no difference in enzyme activity assay between such two types, we just used one name to represent them herein.

**Fig 1:**
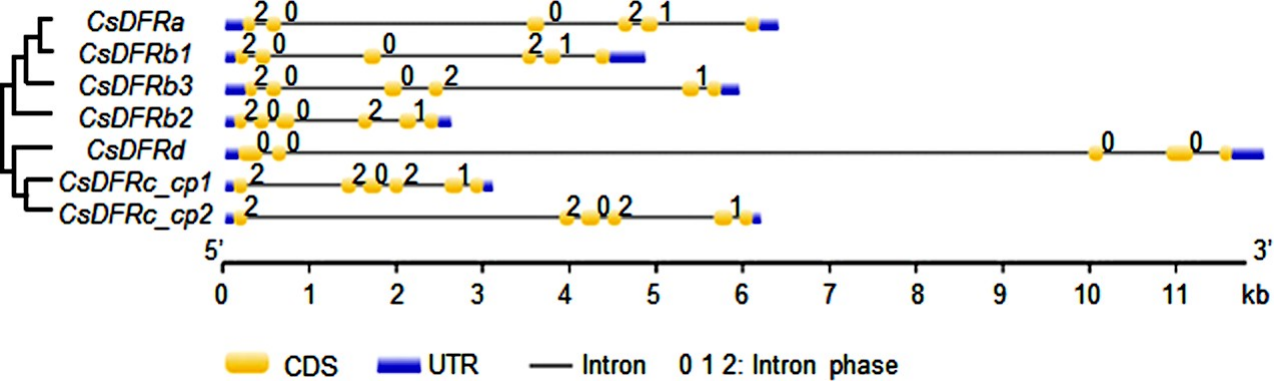
Gene structures of putative *CsDFRs* with phylogenetic relationship of their mRNAs. *CsDFRa* and *CsDFRbs* have the same exon amounts and intron phases. Data for this figure could be found in S2 File.

To display the gene structures of *CsDFRs*, their mRNAs annotated in SCZ genome (Acc. No. in Table 1) were aligned to the genomic DNA and the splicing sites and the sequences of mRNAs were retrieved. Then, the sequencing results of genes cloned by ourselves were mapped to the gene sequences to adjust the splicing. Referring to the alignment results and splicing sites indicated in the original general feature format 3 (gff3) file from the SCZ genome data, some locus coordinates were modified and intron phases were re-calculated to generate a new gff3 file (S2 File). A phylogenetic tree of mRNAs was constructed by MEGA software and the distance information was extracted from the resulting nwk file (S2 File). Information in the new gff and nwk files were input into GSDS to draw a gene structure picture (Fig 1). The result shows that *CsDFRa*, *CsDFRbs* and *CsDFRc* have six exons with nearly the same lengths, while *CsDFRd* harbours one less. *CsDFRc* has two copies (named as *CsDFRc_cp1* and *CsDFRc_cp2*) with the same exon but different intron lengths, which indicates that they may be not allelic. Other partial copies for *CsDFRs* were not considered as functional genes and therefore were not analysed here (neither were the transcripts from intron retention events of *CsDFRb1* [14]). The predicted intron phases of *CsDFRa* and *CsDFRbs* are conserved (i.e. 2, 0, 0, 2, 1), while those of *CsDFRc* and *CsDFRd* are different, but two copies of *CsDFRc* are still identical (i.e. 2, 2, 0, 2, 1). The gene structure analysis indicates that only three *CsDFRbs* have the same exon amounts and intron phases with *CsDFRa*, the function-identified *DFR*.

### Protein sequence analysis of putative CsDFRs

According to the sequencing results of cloned *CsDFRs*, their protein parameters were predicted (Table 1). Six putative CsDFR proteins range from 344 to 357 amino acids (a.a.) in size, with molecular weights (Mol. Wt.) varying from 36.8 to 39.1 kDa, and theoretical isoelectric points (pI) from 5.70 to 7.04, which indicates that CsDFRs, except for CsDFRd, are acidic proteins. The shorter CsDFRa contains 340 a.a. with 38.0 kDa and pI 5.73.

**Fig 2:**
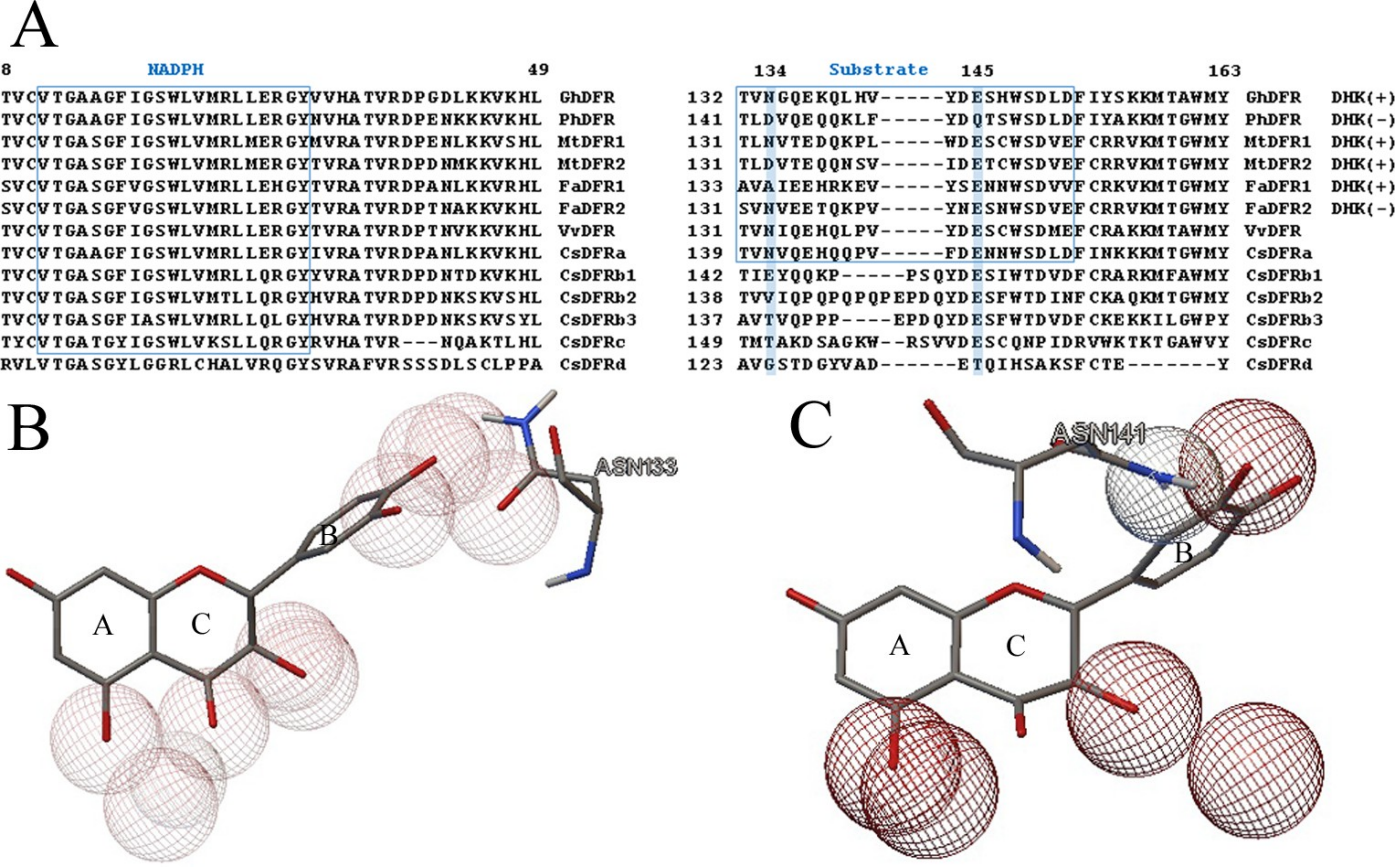
Alignment of amino acid sequences of putative CsDFRs with two types of DFRs (utilizing DHK or not) and interactions between enzyme DFR and substrate DHQ. **(A)** Alignment of amino acid sequences. The numbers on the top indicate residuals of GhDFR, corresponding to those used by Johnson et al. [20]. The box named “NADPH”: NADPH-binding domain. The box named “Substrate”: substrate-specificity-determining region. (+) or (-) for “DHK” on the right of DFR sequences indicates whether these characteried DFRs accept DHK as substrate (Asp-type) or not (Asn-type). The accession numbers of the protein sequences are as follows: Gh (*Gerbera hybrida*): P51105.1; Ph (*Petunia hybrida*): P14720.2; Mt (*Medicago truncatula*): AAR27014.1, AAR27015.1; Fa (*Fragaria x ananassa*; Strawberry): AHL46444.1, AHL46451.1; Vv (*Vitis vinifera*): P93799; Cs (*Camellia sinensis*, see Table 1). **(B)** Interaction between VvDFR and DHQ from Protein Data Bank (PDB): 2C29. Three rings of DHQ were marked as A, C, B, respectively. Hydrogen bonds were displayed as wireframe spheres. **(C)** Interaction between CsDFRa and DHQ. Asn141 equals to Asn133 in VvDFR and N134 in GhDFR. More details could be referred to S1 Fig.

Multiple sequence alignment of CsDFR proteins was performed by DNAMAN software. Generally, the deduced CsDFR proteins contain conserved NADPH-binding domains (except for CsDFRd), resembling the NAD-dependent epimerase/dehydratase family [18]. But only CsDFRa harbours conserved substrate-specificity-determining region like DFRs in other plants (Fig 2A). This indicates that maybe CsDFRa is unique. The asparagine residual at position 134 (GhDFR numbering, i.e., N133 of VvDFR in Fig 2B and N141 of CsDFRa in Fig 2C) is said to be important in preferring substrate. Thus, CsDFRa could be classified into Asn-type DFRs which convert DHK inefficiently [31]. This is in accordance with the fact that pelargonidin-based anthocyanins are barely detected in tea plant. The residual of this site in CsDFRb1 is E, whose property is similar to D. Thus, CsDFRb1 may belong to Asp-type. The remaining putative CsDFRs are neither Asn- nor Asp-type.

**Fig 3:**
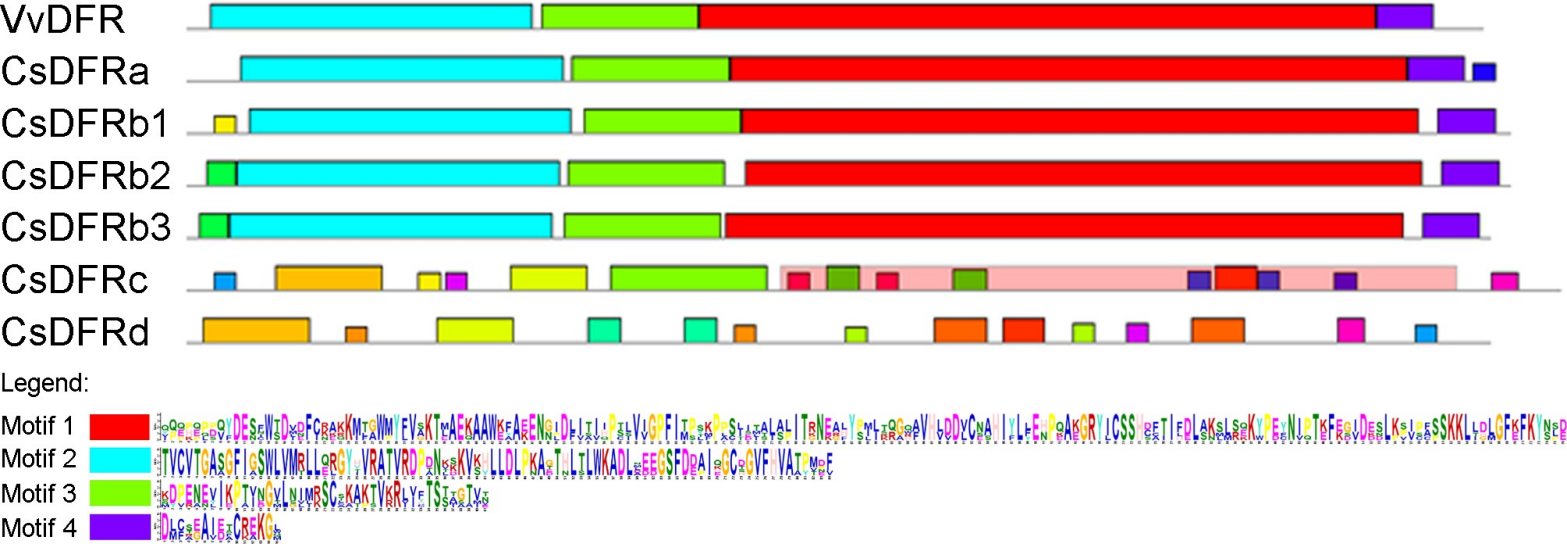
Conserved motifs of putative CsDFR proteins (VvDFR was also displayed as a reference). The colored boxes represent different motifs and their position in proteins. Out of 20 conserved motifs analyzed by MEME, four motifs are significant (E-value < 0.01) and illustrated in the legend.

The conserved motifs in CsDFR proteins were further analyzed on the website of Multiple Em for Motif Elicitation (MEME), and four putative motifs were significantly retrieved (Fig 3). After searching the motifs on the HMMER website, it was annotated that Motif 1 (142–317 a.a. in CsDFRa numbering) corresponded to NAD dependent epimerase/dehydratase family; Motif 2 (15–98 a.a.) encoded a NAD(P)H-binding domain; Motif 3 (101–141 a.a.) and Motif 4 (318–332 a.a.) did not match any functional annotation. VvDFR was also analyzed as a reference. CsDFRa and CsDFRbs contained the above four conserved motifs, just like VvDFR.

Furthermore, through the analysis of the amino acid sequences of CsDFRs, we found that transmembrane helices (predicted by TMHMM) existed in CsDFRa (7–29 a.a.) and CsDFRb1 (10–32 a.a.), which were not common in other plant DFRs investigated herein, except for PhDFR (10–32 and 195–217 a.a.) and GhDFR (7–24 a.a.). In addition, the subcellular location of CsDFRa was predicted in chloroplast but this prediction was not reliable, as its reliability class (RC) was 5. Meanwhile, CsDFRd was predicted in mitochondrion (RC = 1), and the remaining putative CsDFRs were supposed in other locations (except chloroplast, mitochondrion and secretory pathway).

**Fig 4:**
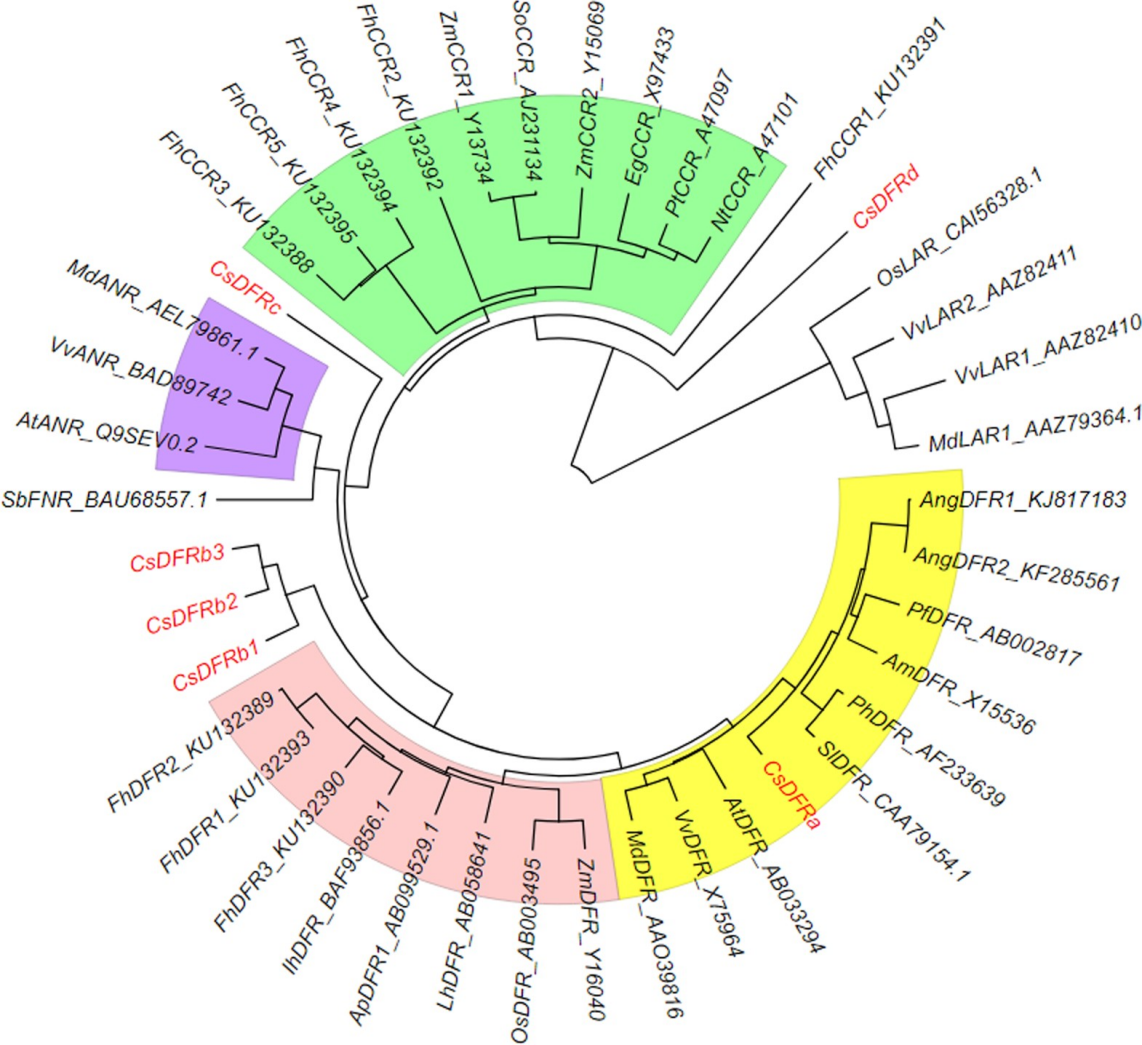
Phylogenetic tree of putative CsDFR proteins with other similar proteins. Protein names and accession numbers are marked on each node, according to Li et al. [16]. Yellow: Dicotyledon DFR; Red: Monocotyledon DFR; Blue: ANR (anthocyanidin reductase); Green: CCR (cinnamoyl-CoA reductase).

A phylogenetic tree of putative CsDFR proteins was constructed with their full lengths of amino acid sequences to investigate the evolutionary relationships among DFRs (Fig 4). Because CsDFRc and CsDFRd are similar to other reductases, the tree also included some anthocyanidin reductases (ANR), cinnamoyl-CoA reductases (CCR), flavanone 4-reductases (FNR) and leucoanthocyanidin reductases (LAR). The result proved that CsDFRa was the only DFR in the tea plant that had a close relationship with other dicotyledonous plants. Three CsDFRbs formed a distinctive branch, far from both monocotyledonous and dicotyledonous plants. CsDFRc and CsDFRd did not belong to a clear subgroup either. In view of the ambiguous origin of these putative CsDFRs (other than CsDFRa), it seemed necessary to identify their real function.

### Identification of enzyme function

**Fig 5:**
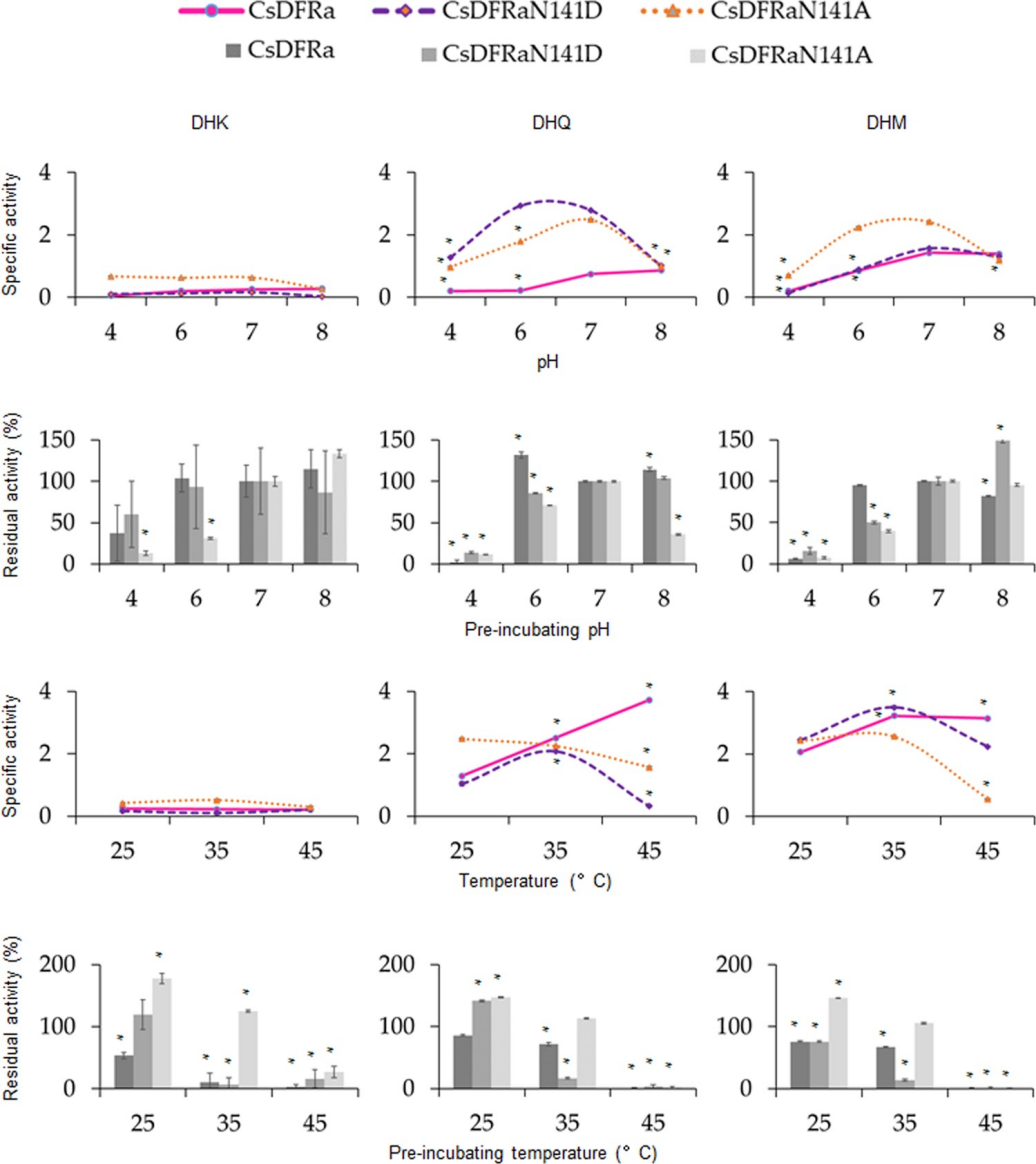
Optimal temperatures and pHs (line charts) and thermal and pH stability (bar charts) of CsDFRa and its two mutants. * The value is significantly different (*p* < 0.05) compared to the corresponding control (pH 7 and 25°C were the control for pH and temperature, respectively, and their results, which were not pre-incubated, were deemed as 100% activity in the enzyme stability assay).

To identify the catalytic function of putative CsDFR enzymes on reducing DHFs, prokaryotic expression and protein purification were implemented. In addition, we constructed two mutants with a single amino acid changed at position 141 of CsDFRa. The natural Asn (N) was substituted by Asp (D) or Ala (A). Firstly, the optimal temperature and pH, and the thermal and pH stability of CsDFRs were examined, together with the effect of single amino acid mutation on CsDFRa’s characteristics (Fig 5). The results revealed that only CsDFRa and its two mutants exhibited enzyme activity on DHFs, while other putative CsDFRs showed little activity under these conditions. The optimum pH for the activities of CsDFRa, CsDFRaN141D and CsDFRaN141A were all around 7. The three enzymes were not stable in an acidic solution, especially at pH 4, where the remaining activities were less than 10%. The wild type CsDFRa was robust at pH 6 for both DHQ and DHM, whereas the two mutants were not. Enzymes kept active at pH from 7 to 8 except for the mutation N141A, whose catalytic ability for DHQ was decreased to 36% of neutral pH ability. In the temperature experiment, CsDFRa’s activity for DHQ rose with the increasing reaction temperature. But it rose no more for DHM with temperature beyond 35°C. This again proved that natural CsDFRa could catalyze well with DHQ, which also indicated that at high temperatures, tea plant might generate more metabolites from DHQ than from DHM. Two mutants were most active at 35°C and declined at 45°C. The single residual mutation might have influenced the stability of CsDFRa. However, all three enzymes lost their ability after being pre-incubated at 45°C for half an hour. In the mutation N141D, activity was weakened to about 15% of original for both DHQ and DHM at 35°C. Meanwhile, no conditions could promote CsDFRa’s activity on DHK.

**Fig 6:**
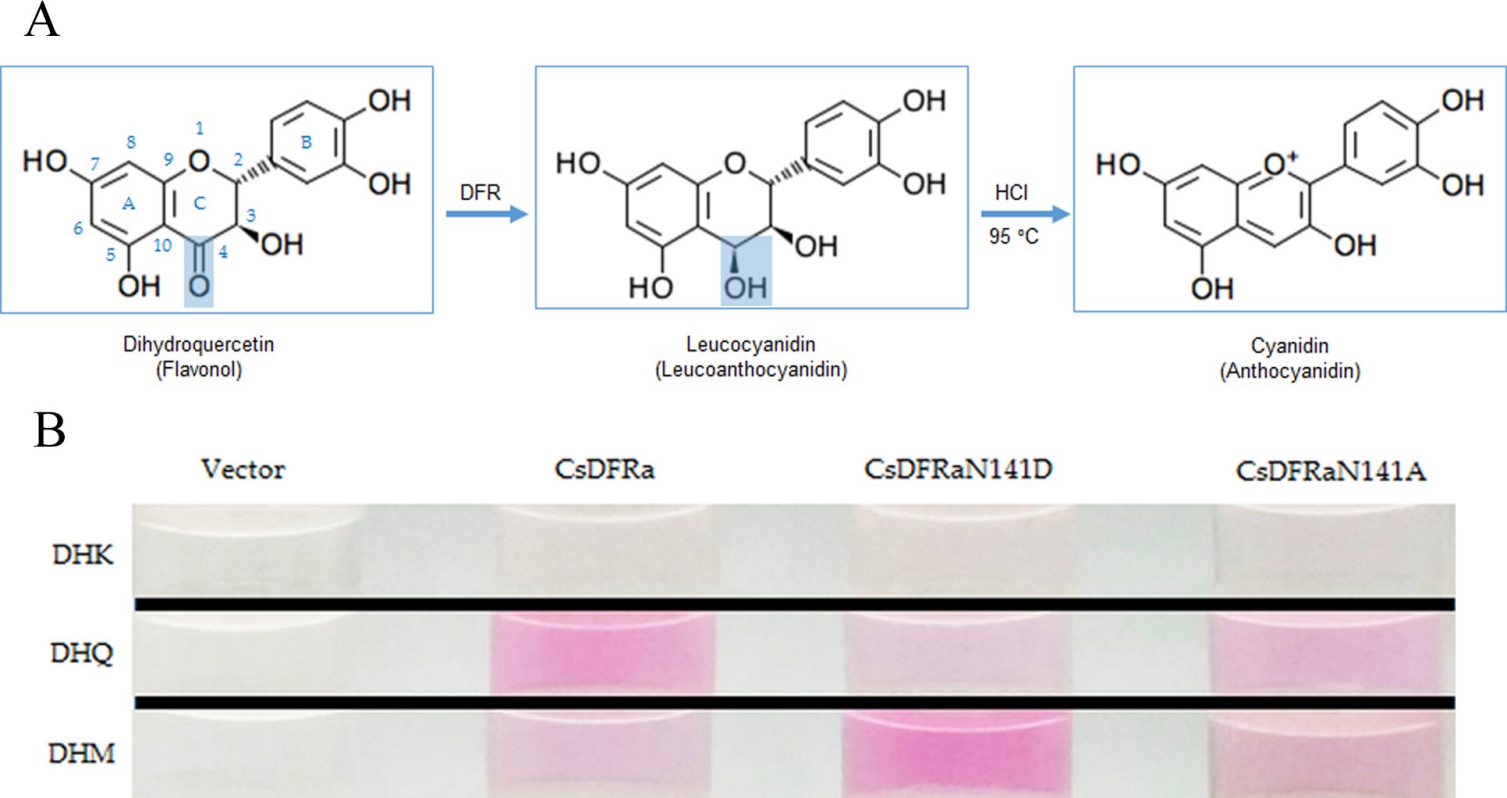
Anthocyanidins generated from leucoanthocyanidins produced by prokaryotic expressed CsDFRa and its mutants. **(A)** A schematic diagram depicts the reaction process. **(B)** Anthocyanidin products in reaction tubes. Among six putative CsDFRs, only CsDFRa exhibited enzyme activity on DHFs, while other five CsDFRs’ results were similar to that of the control group which was added with the protein expressed by the pET-32a vector.

DFR catalyzes the reduction of C4 in the C ring of DHFs to form leucoanthocyanidins (Fig 6A). Since the DFR products, leucoanthocyanidins, are colorless, we converted them into corresponding anthocyanidins by incubating the products at 95°C in acidic alcohols (Fig 6A), which equaled to the function of anthocyanidin synthase (ANS) in plants. The enzyme function was then determined based on color change. Still, only CsDFRa generated colored products, while other five putative CsDFRs’ results were not different from the control group. Among the CsDFRa’s products, the one generated from DHQ looked deepest (A_520_ = 0.693±0.031), while the one from DHK seemed very little (A_520_ = 0.038±0.010) (Fig 6B). Interestingly, an anticipated increased activity of two CsDFRa mutants on DHK according to previous references [10,32] was not observed. Instead, it was found that such substitutions led to changes in preference for DHQ and DHM. CsDFRaN141D seemed to utilize DHM most (A_520_ = 0.745±0.026) while CsDFRaN141A catalyzed DHQ and DHM evenly (A_520_ = 0.453±0.023 and A_520_ = 0.412±0.027, respectively). This was consistent with the above-mentioned investigation on enzyme kinetics.

To investigate the characteristics of CsDFRs, the decrease of NADPH was determined to define the enzyme activity. Again, CsDFRb1~CsDFRd showed very little activity. And two length types of CsDFRa (S1 File) showed no difference in their function. Thus, only CsDFRa and its mutants were then studied. Table 2 shows the kinetic parameters of CsDFRa for three DHF substrates. The reaction rate was faster for DHM (*V*_max_ = 1.55 nmolNADPH/min·μgProt). However, due to higher affinity for DHQ (the lowest *K*_m_ = 8.0 μM), the final catalytic efficiency (*K*_cat_/*K*_m_) of CsDFRa was almost 3-fold higher for DHQ than that for DHM. The mutation of N141D attenuated the enzyme affinity with DHQ, which resulted in nearly 7-fold higher efficiency for DHM than that for DHQ. The mutation of N141A had the same rate and affinity for both DHQ and DHM. All three enzymes exhibited little effects on DHK.

**Table 2:** Kinetics of CsDFRa and its two mutants for three DHF substrates.

Enzyme	*V*_max_ (nmolNADPH/min μgProt)			*K*_m_ (μM)			*K*_cat_/*K*_m_ (L/kg*s)		
	DHK	DHQ	DHM	DHK	DHQ	DHM	DHK	DHQ	DHM
CsDFRa	0.34	0.82	1.55	539.8	8.0	43.0	10.6	1718.2	601.7
CsDFRaN141D	0.09	2.96	3.00	-	252.0	37.2	-	195.8	1344.1
CsDFRaN141A	1.94	4.40	4.33	398.1	88.5	90.6	81.2	829.2	797.4

### Subcellular localization

**Fig 7:**
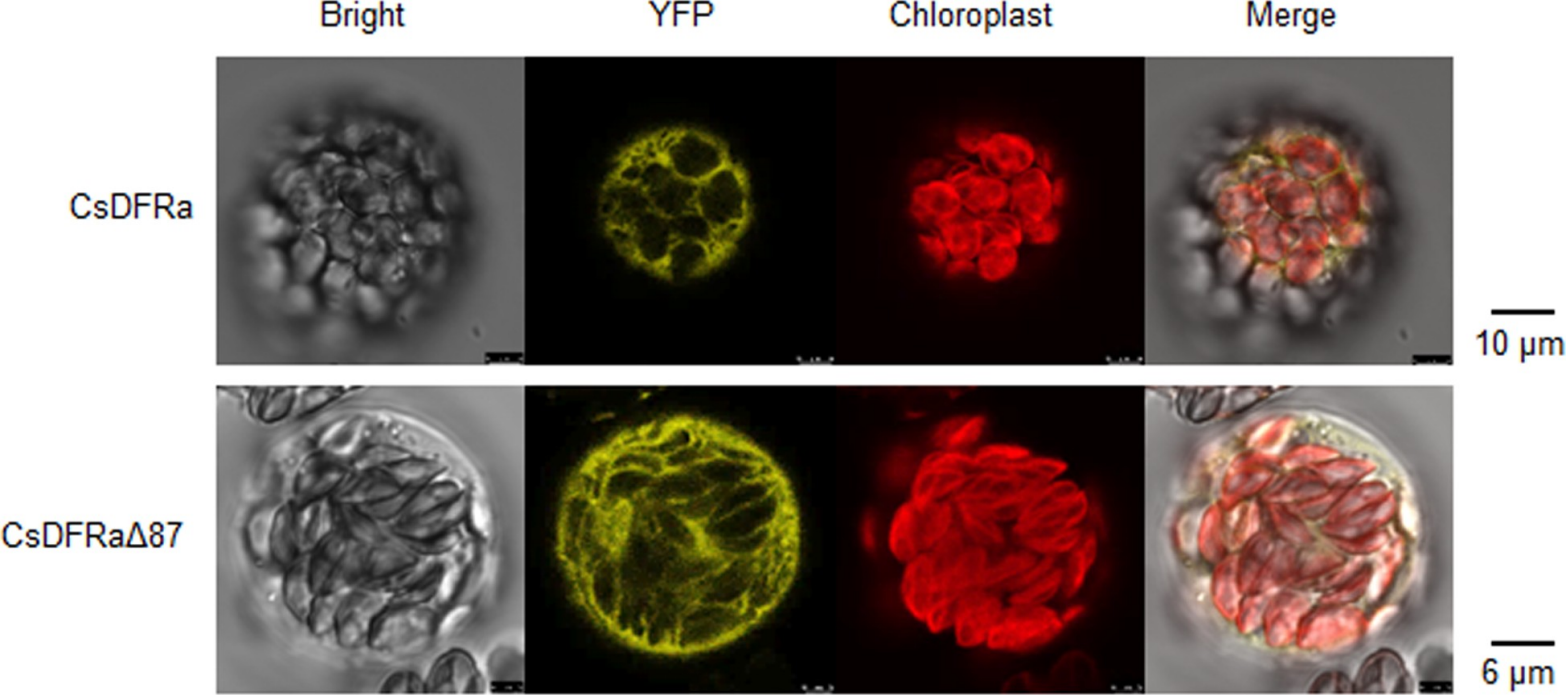
Subcellular location of CsDFRa and CsDFRaΔ87.

Furthermore, considering the transmembrane domain at N-terminal (1~29 residuals) of CsDFRa, subcellular localization of CsDFRa with or without (named CsDFRaΔ87) this domain was detected by constructing the two genes fused with yellow fluorescence protein, transforming them into *Arabidopsis thaliana* protoplasts. The results proved that both CsDFRa and CsDFRaΔ87 were localized in cytoplasm (Fig 7).

### Promoter analysis and expression profile of putative *CsDFRs*

**Fig 8:**
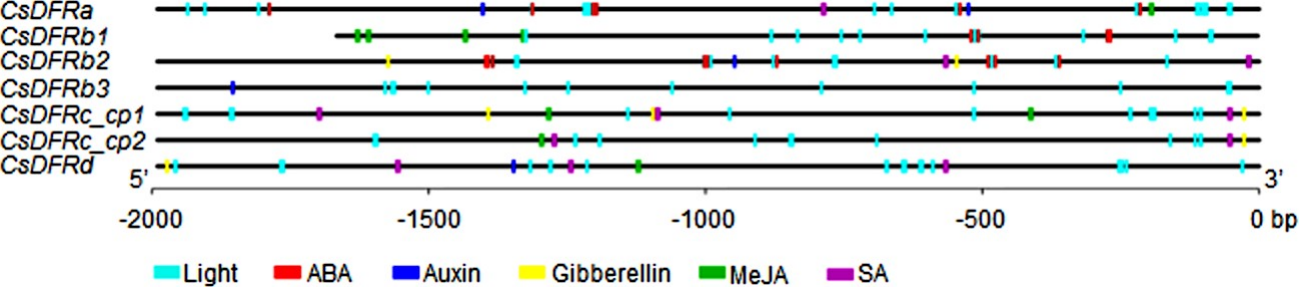
Promoter analysis of *CsDFR* genes. Black lines indicate promoter regions. Cis-acting elements involved in response to light and phytohormones are represented by color boxes.

To further understand the regulation and behavior of putative *CsDFRs*, 2 Kb of promoter sequences (for *CsDFRb1*, the length was 1674 bp) were submitted to PlantCARE website and cis-elements were searched. There are 259 cis-elements with 30 kinds in *CsDFRa*’s promoter region, which are the most among all putative *CsDFRs*. For other *CsDFRs*, 85~187 elements with 16~29 kinds were found (S2 File). The 300 bp upstream sequences of *CsDFRc_cp1* and *CsDFRc_cp2* have 95% similarity. All these promoters, especially *CsDFRa*, are rich in light-responsive elements, such as G-Box, I-Box, Box 4, etc.. *CsDFRa*, *CsDFRb1* and *CsDFRb2* also have several ABA-responsive elements. In addition, cis-elements involved in seed specific expression were only found in *CsDFRa*’s promoter. The distribution and numbers of Cis-elements responding to phytohormones and stresses were displayed in Fig 8 and Table 3, respectively.

**Table 3:** Counts of cis-elements of putative *CsDFRs* responding to hormones and stresses.

Signal	*CsDFRa*	*CsDFRb1*	*CsDFRb2*	*CsDFRb3*	*CsDFRc_cp1*	*CsDFRc_cp2*	*CsDFRd*
ABA	11	6	12	0	0	0	0
Auxin	2	0	1	1	0	0	1
Gibberellin	0	0	3	0	3	1	1
MeJA	2	8	0	0	4	2	2
SA	1	0	2	0	3	2	3
Light	19	13	13	11	10	10	14
Anoxia	2	0	6	4	0	4	1
Oxidative	1	7	1	1	2	3	1
Wound	2	5	2	0	0	1	2

**Fig 9:**
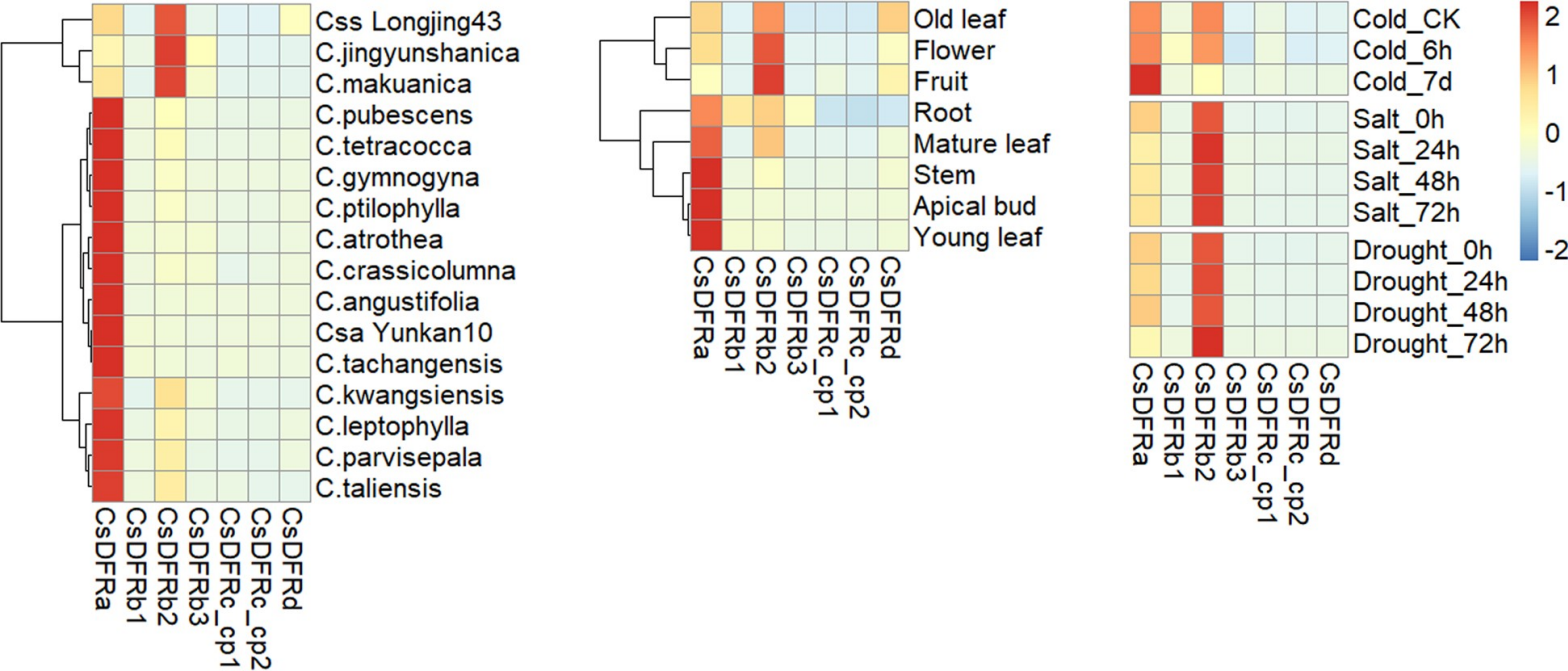
Expression profile of putative *CsDFRs* among various varieties, tissues and stresses.

Expression data were extracted from TPIA (Tea Plant Information Archive) website (accession IDs were shown in Table 1). Generally, expression of *CsDFRa* and *CsDFRb2* was higher than that of other genes in various *Camellia* species and tissues (Fig 9). Expression of *CsDFRa* was higher in big-leaf species (Csa var. Yunkan10) than that in small-leaf species (Css var. Longjing43). However, the expression profile of *CsDFRb2* was opposite. Few fragments were detected for other genes. The expression levels of *CsDFRa* were the highest, even 10-fold higher than *CsDFRb2*, in apical bud, young and mature leaves, and stem. Three *CsDFRbs* were highly expressed in root. In general, expression of all *CsDFRs* decreased slightly under cold, salt and drought stresses, while *CsDFRa* and *CsDFRb2* were slightly promoted by cold or salt/drought stimulation at some time points, respectively.

## Discussion

DFR is an important gene correlated with proanthocyanins in the leaf [33] and pigmentation in flower [13]. Deactivation of DFR decreased anthocyanins [34]. It is a key regulatory point controlling the carbon flux into distinct anthocyanins. In previous publications, several *CsDFR* members were discussed as they were annotated as DFR genes in transcriptome data [13–15]. However, so far, all the papers relating to *CsDFR* identification focused on one isoform [17–19]. Regarding the important role and extensive discussion of *CsDFR*, we felt that it was necessary to make clear the function of those putative *CsDFRs*.

Through the analysis of gene sequences, we found that three *CsDFRbs* had some similarity with *CsDFRa*, while *CsDFRc* and *CsDFRd* were more like cinnamyl-alcohol dehydrogenase and short-chain dehydrogenases/reductases, respectively. *CsDFRa*, *CsDFRbs* and *CsDFRc* have 6 exons, and *CsDFRa* and *CsDFRbs* have the same intron phases. The gene structures of *CsDFRa* and *CsDFRbs* are the same as the *DFR* from *Populus trichocarpa* [35].

Analysis of protein sequences and structures of putative CsDFR proteins proved that CsDFRa was the most conserved DFR in tea plant compared to those in other plants. In the protein Blast results, CsDFRb1 is similar to the DFR (Acc. No. PSR99659.1) from *Actinidia chinensis* var. chinensis, while CsDFRb2 and CsDFRb3 are similar to PSR99661.1. CsDFRc and CsDFRd are similar to PSS16240.1 and PSR99760.1 from *Euphorbia pulcherrima*. But the function of these DFR in other plants have not yet been identified. The CsDFRa cloned from our Baitang purple tea variety is the same as the CsDFR of Yabukita variety (Acc. No. AB018685.1), and has a two amino acid difference (D159H, V202G) from CsDFR of UPASI-10 variety (Acc. No. AY648027) in India [18,36], and one difference (E99K) from CsDFR of Line 2043 variety (Acc. No. AY574920) in Sri Lanka [17]. Because only one copy of *CsDFRa* was detected in genome data, its two types of C-terminal may be not resulted from alternative splicing, but rather due to a mutation. The published CsDFRs in other tea varieties (all cloned from 3’ UTR and sequenced), and the sequence retrieved from CSA genome data are the longer type. The short type is only discovered in CSS genome data and our variety. For that reason, our Baitang purple tea might be a hybrid of CSS and CSA.

According to the N-terminal sequence, the subcellular location of CsDFRa was predicted in chloroplast by SignalP but with low reliability. It has been reported that CsDFRa was localized in cytoplasm of transiently expressed tobacco leaves [14], and VbDFR from *Vitis bellula* also showed the cytosolic localization in onion epidermal cells [37]. We predicted transmembrane structure was in the N-terminal of CsDFRa, which is from 1 to 29 amino acid residues. Herein, we constructed recombinant plasmids containing YFP and *CsDFRa* with or without transmembrane domain (*CsDFRaΔ87*), and transformed them into *Arabidopsis thaliana* protoplasts individually. The results confirmed that CsDFRa was in cytoplasm, but little difference was detected between CsDFRa and CsDFRaΔ87 (Fig 7).

Promoter analysis revealed that *CsDFRa* had more cis-elements than other putative *CsDFRs*, suggesting that *CsDFRa* might be the predominant DFR in tea plant responding to internal and external cues. Moreover, only Cs*DFRa*’s promoter has elements for seed specific expression. It was considered that high expression of DFR in seeds was consistent with accumulation of proanthocyanins and leucoanthocyanidins [18]. Some elements in *CsDFRa* promoter have been characterized, such as W-box [38] and E-box [39]. The expression levels of *CsDFRa* have been widely reported in previous papers. It reached highest in buds and first leaves [40], but decreased when they are in shade [33]. Meanwhile, epicatechins declined and catechins increased, indicating that *CsDFRa* is closely and positively correlated with epicatechins which are generated through anthocyanidins [41]. For purple tea, *CsDFRa* in 2~3 leaves (purple) was 2-folder higher than that in 4~5 leaves (green) of Zijuan variety [42]. CsDFR is usually downregulated under stresses, such as drought [18] and low temperature [43]. Other putative *CsDFRs* showed the same trends, but their expression levels were very low [13–15]. The expression profiles of *CsDFRs* revealed in this study coincide with those previous publications. In spite of the smaller increasing folders (about 2~3 folders) of DFR compared to other genes like ANR in the above situations, DFR acts somewhat like a valve in flavanoid metabolism. Its preference controls the carbon flux from DHFs into different branches of anthocyanidins and even catechins.

Referring to the substrate specificity, it is quite different for DFRs from diverse species. It was reported that PhDFR with D134 (Gerbera numbering, Asp at 134 site, equalling to N133 of VvDFR, the same below) cannot catalyze DHK, while many other DFRs with N134 can use DHK as substrate [20]. We compared all nine residuals interacting with DHQ (PDB Entry: 2C29) [21], and found that only one mutation of N134 makes MtDFR1 prefer DHK more than MtDFR2 (D134) [10]. Also in *Fragaria* species, one mutation in FaDFR1 (A134) compared to FaDFR2 (N134), results in higher affinity for DHK [32]. From the crystal structure, we could see that when the substrate is DHQ (two hydroxyl groups in B-ring), there are three hydrogen bonds fixing to the B-ring (S1 Fig; The same with myricetin as substrate as shown in PDB Entry 2IOD). However, DHK only has one hydroxyl group in the B-ring, which will surely decrease hydrogen bonding. Moreover, if N134 was substituted by D134, the oxygen in the hydroxyl group of B-ring could not form a hydrogen bond. We supposed that the affinity would be weaker. On the other side, the polarity of DHK’s B-ring is lower than DHQ’s and DHM’s, and the hydrophobicity is stronger. We assume that A134, together with A130 and I223 (i.e. Ala129 and Ile222 of *Vitis vinifera*), provide a hydrophobic environment for DHK’s B-ring, which may then make it more stable than N134 does. A similar speculation was proposed for the N133L mutant in GhDFR, which may prevent binding of DHFs due to the bulky and nonpolar leucine residue [21]. In this study, however, two mutations in the corresponding position of CsDFRa (N141D and N141A) did not help it catalyse DHK as we had expected. Furthermore, it was said that this mutation would not change the H-bond network and had no substrate selectivity [21]. However, what we found here demonstrated this mutation in CsDFRa could adjust its preference for DHQ and DHM. We realized from the crystal structure that a VvDFR unit could adopt two DHMs, which makes the substrate-preference more complicated. This proved the view that single amino acid mutation at this site was important but not sufficient to explain the preference of DFR for substrates. Furthermore, CsDFRa was the only functional one among the six enzymes, and had similar *K*_m_ value with *Medicago truncatula* [10], but larger than those from *Fragaria* species [32]. However, the final efficiency of CsDFRa was dozens of times higher. Due to the ambiguous function of *CsDFRs* except for *CsDFRa*, their sequencing results have not been uploaded to NCBI yet, and *CsDFRa* has many accession numbers already. More substrates need to be tested and *in planta* experiment could be implemented in future to unveil the potential function of the five putative reductases, especially for the relatively highly expressed *CsDFRb2*.

Taken together, the other five putative *CsDFR* genes were very different from *CsDFRa*. *CsDFRbs* may function on substrates with structures similar to DHFs [17], but further investigation is necessary. This study elucidated that only one CsDFR plays a role in the pathway to produce anthocyanidins and catechins, which will make omics analysis more accurate in future. Furthermore, we have elucidated some of the regulating mechanisms of CsDFR with regards to structure, promoter, enzyme nature and so on. The discovery of the characteristics of two artificial DFR mutants shed light on a possible direction for future screening or modification of tea germplasm resources with different composition of anthocyanins or catechins.

## Supporting information

S1 Fig Molecular docking results of DFR with DHQ and DHM. Two DHM molecules, named as MYC4341 and MYC4342 in the template of 2IOD.pdb, were herein represented as DHM_1 and DHM_2, respectively.
(TIF)

S1 File Nucleotide, amino acid and promoter sequences of putative CsDFRs. (DOC)

S2 File Primers and raw data for drawing. (XLSX)
