# Supplementary figures and images for: Comprehensive analysis of putative dihydroflavonol 4-reductase gene family in tea plant
Source: PLoS One. 2019 Dec 26;14(12):e0227225. doi: 10.1371/journal.pone.0227225 (PMC6932780; doi:10.1371/journal.pone.0227225)

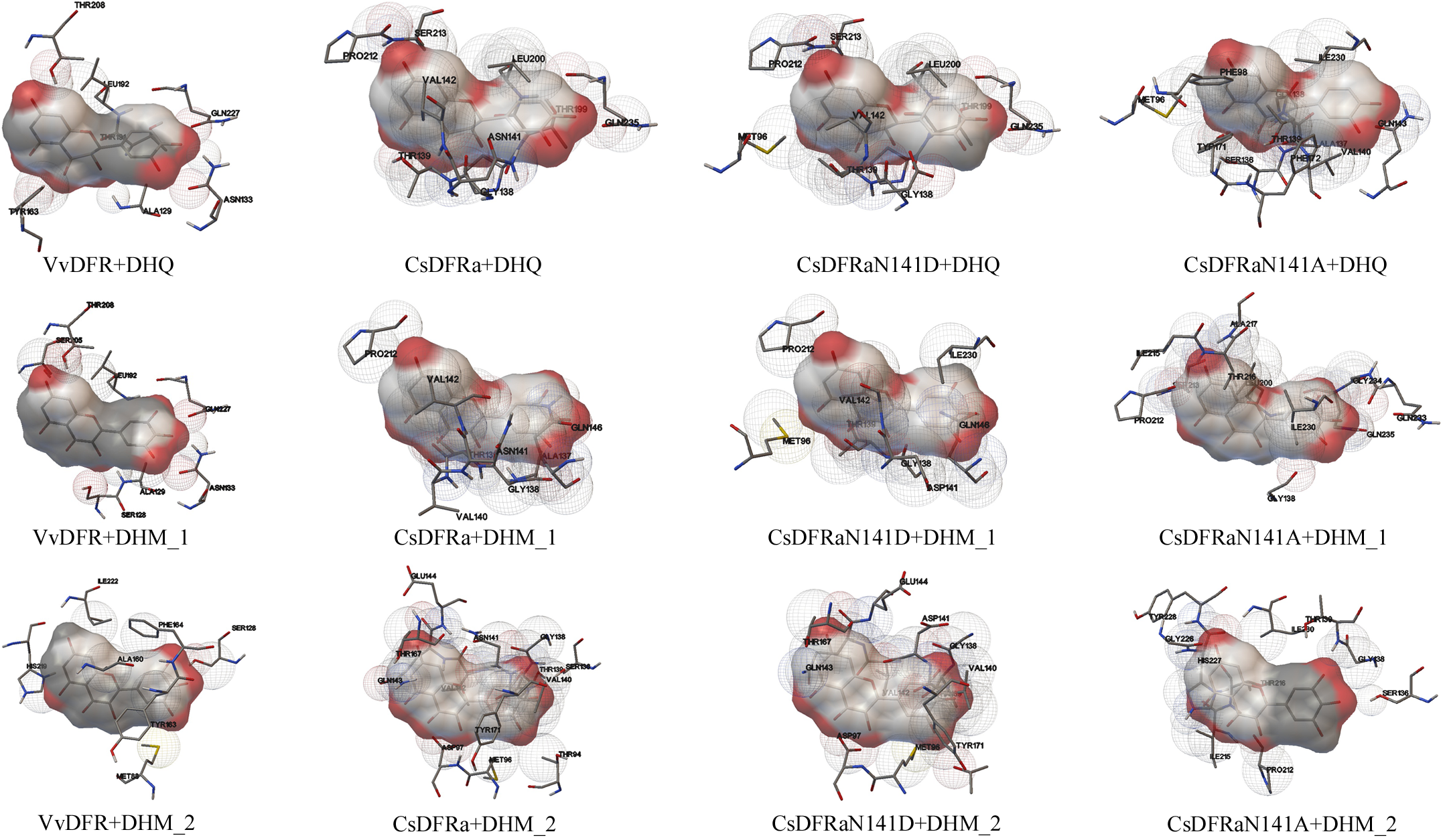

Supplement: S1 Fig — Two DHM molecules, named as MYC4341 and MYC4342 in the template of 2IOD.pdb, were herein represented as DHM_1 and DHM_2, respectively. (TIF) [file pone.0227225.s001.tif]
