# Supplementary material for: Comprehensive analysis of putative dihydroflavonol 4-reductase gene family in tea plant
Source: PLoS One. 2019 Dec 26;14(12):e0227225. doi: 10.1371/journal.pone.0227225 (PMC6932780; doi:10.1371/journal.pone.0227225)
Supplement: S1 File — (DOC) [file pone.0227225.s002.doc]

S1 File: sequences of putative *CsDFRs*

Contents

[1. DNA-sequencing results of putative *CsDFRs* cloned from Baitang purple tea leaves 1](#__RefHeading___Toc22149499)

[>CsDFRa_long 1](#__RefHeading___Toc22149500)

[>CsDFRa_Short 2](#__RefHeading___Toc22149501)

[>CsDFRb1 2](#__RefHeading___Toc22149502)

[>CsDFRb2 2](#__RefHeading___Toc22149503)

[>CsDFRb3 3](#__RefHeading___Toc22149504)

[>CsDFRc 3](#__RefHeading___Toc22149505)

[>CsDFRd 3](#__RefHeading___Toc22149506)

[2. Amino acid sequences of putative CsDFRs 4](#__RefHeading___Toc22149507)

[>CsDFRa_long_38.70kDa 4](#__RefHeading___Toc22149508)

[>CsDFRa_short_37.97kDa 4](#__RefHeading___Toc22149509)

[>CsDFRb1_38.5kDa 4](#__RefHeading___Toc22149510)

[>CsDFRb2_38.47kDa 4](#__RefHeading___Toc22149511)

[>CsDFRb3_37.54kDa 4](#__RefHeading___Toc22149512)

[>CsDFRc_39.09kDa 4](#__RefHeading___Toc22149513)

[>CsDFRd_36.85kDa 5](#__RefHeading___Toc22149514)

[3. Promoter sequences of putative *CsDFRs* extracted from SCZ genome data 5](#__RefHeading___Toc22149515)

[>CsDFRa_promoter 5](#__RefHeading___Toc22149516)

[>CsDFRb1_promoter_1674bp 5](#__RefHeading___Toc22149517)

[>CsDFRb2_promoter 6](#__RefHeading___Toc22149518)

[>CsDFRb3_promoter 7](#__RefHeading___Toc22149519)

[>CsDFRc_cp1_promoter 7](#__RefHeading___Toc22149520)

[>CsDFRc_cp2_promoter 8](#__RefHeading___Toc22149521)

[>CsDFRd_promoter 8](#__RefHeading___Toc22149522)

1. DNA-sequencing results of putative *CsDFRs* cloned from Baitang purple tea leaves

>CsDFRa_long

ATGAAAGACTCTGTTGCTTCTGCCACAGCCTCCGCACCGGGCACCGTGTGTGTCACCGGAGCCGCTGGATTCATCGGCTCGTGGCTCGTCATGAGGCTGCTCGAACGCGGCTATATTGTTCGTGCAACCGTTCGCGATCCAGCGAATTTAAAGAAGGTGAAGCACTTGTTAGACTTGCCGAAAGCTGACACGAACTTGACACTGTGGAAGGCGGATTTGAATGAAGAAGGGAGCTTTGATGAGGCCATTGAGGGTTGCTCTGGAGTGTTTCATGTTGCCACACCTATGGATTTTGAGTCTAAGGACCCTGAGAATGAGGTAATCAAGCCGACAATCAATGGTGTGTTGAGCATCATAAGGTCATGCACCAAAGCTAAGACAGTGAAGAGGCTGGTGTTCACATCCTCTGCTGGAACTGTTAATGTCCAGGAACACCAACAACCCGTTTTCGACGAGAACAATTGGAGTGACTTGGATTTCATCAATAAGAAGAAGATGACTGGCTGGATGTATTTTGTTTCAAAAACATTGGCAGAGAAAGCAGCATGGGAAGCAGCAAAAGAGAACAACATTGATTTCATTAGTATCATTCCTACATTAGTTGTAGGACCTTTCATAATGCCAACATTCCCACCAAGCCTAATCACTGCTCTCTCCCCCATCACTAGGAATGAAGGACACTACTCGATCATAAAGCAAGGGCAGTTTGTGCACCTTGATGATCTCTGTGAATCTCATATATTCTTGTATGAGCGTCCTCAGGCTGAGGGCAGATACATTTGCTCCTCCCATGATGCTACCATCCATGATTTGGCCAAACTGATGAGAGAGAAATGGCCCGAGTACAATGTCCCCACTGAGTTTAAGGGGATAGATAAGGACTTGCCAGTTGTGTCGTTCTCATCGAAGAAGTTGATAGGAATGGGGTTTGAATTCAAGTATAGCTTGGAGGACATGTTCAGAGGAGCCATTGATACTTGCAGAGAGAAGGGTTTGCTTCCTCACTCTTTTGCAGAAAACCCTGTCAATGGCAACAAGGTTTAA

>CsDFRa_short

CACATTTTAATCCCATCATGAAAGACTCTGTTGCTTCTGCCACAGCCTCCGCACCGGGCACCGTGTGTGTCACCGGAGCCGCTGGATTCATCGGCTCGTGGCTCGTCATGAGGCTGCTCGAACGCGGCTATATTGTTCGTGCAACCGTTCGCGATCCAGCGAATTTAAAGAAGGTGAAGCACTTGTTAGACTTGCCGAAAGCTGACACGAACTTGACACTGTGGAAGGCGGATTTGAATGAAGAAGGGAGCTTTGATGAGGCCATTGAGGGTTGCTCTGGAGTGTTTCATGTTGCCACACCTATGGATTTTGAGTCTAAGGACCCTGAGAATGAGGTAATCAAGCCGACAATCAACGGTGTGTTGAGCATCATAAGGTCATGCACCAAAGCTAAGACAGTGAAGAGGCTGGTGTTCACATCCTCTGCTGGAACTGTTAATGTCCAGGAACACCAACAACCCGTTTTCGACGAGAACAATTGGAGTGACTTGGATTTCATCAATAAGAAGAAGATGACTGGCTGGATGTATTTTGTTTCAAAAACATTGGCAGAGAAAGCAGCATGGGAAGCAGCAAAAGAGAACAACATTGATTTCATTAGTATCATTCCTACATTAGTTGTAGGACCTTTCATAATGCCAACATTCCCACCAAGCCTAATCACTGCTCTCTCCCCCATCACTAGGAATGAAGGACACTACTCGATCATAAAGCAAGGGCAGTTTGTGCACCTTGATGATCTCTGTGAATCTCATATATTCTTGTATGAGCATCCTGAGGCTGAGGGTAGATACATTTGCTCCTCCCATGATGCTACCATCCATGATTTGGCCAAACTGATGAGAGAGAAATGGCCCGAGTACAATGTCCCCACTGAGTTTAAGGGGATAGATAAGGACTTGCCAGTTGTGTCGTTCTCATCGAAGAAGTTGATAGGAATGGGGTTTGAATTCAAGTATAGCTTGGAGGACATGTTCAGAGGAGCCATTGATACTTGCAGAGAGAAGGGTTTGCTTCCTCACTCTTTTGCAGAAAACTAATGGCAACAAGG

>CsDFRb1

CCCTAAACTTCTCAACTTCTCTCTCTCTCTCGCAATGGAAGTAGTGGAAGTGAAGAATGGTTATGGTGGTGGTGGTGGAACCACCGTGTGCGTGACCGGAGCTTCAGGTTTCATCGGTTCATGGCTCGTTATGCGTCTTCTTCAACGTGGCTACTATGTCCGAGCCACAGTTCGCGATCCTGATAACACAGACAAGGTTAAGCATCTCTTGGACTTGCCCAATGCTACCACACACCTCAGCTTGTGGAAGGCAGATCTCGATGAAGATGGAAGCTTTGACGATGCCATTCAAGGCTGTCATGGTGTCTTCCACGTCGCCACTCCCATGAATTTCGTTTTTGTCATGGACCCTGAGAATGAGTATATTAAACCGACGGTAGATGGGGTTTTGAACGTAATGAGATCATGCTGCAAGGCCAAGACTGTGAAGAGGATCATATACACATCAACCATGGCAACTATTGAATACCAACAGAAACCCCCTTCCCAATATGATGAGAGTATTTGGACCGATGTGGATTTCTGCAGGGCTCGCAAGATGTTTGCATGGATGTATCTTGTGGCAAAAACAGAGGCTGAGAAAGCTGCATGGAAATTCGCAGAAGAGAACGGCCCTGATCTCATCACCGTCCACCCCTGTAGTGTGATTGGACCATTCATTACACCATACAAGCCTCCCTGCACTTCAATGGCACTTGCCTTAATCACTAAAAATAAAGCTTTTTACCCAATGCTGACCCAAGGACATGCCGTACACGTGGATGATGTGTGCAACGCTCACATCTACTTGTTCGAGCATCCACAAGCTAAAGGAAGATACATTTGCTCATCTCACTCTTTTACCATATTCGATCTTGCTAACTCATTGAGCAAGAAATACCCAGAGTACAACATTCAGACCAAATTTGAGGACATTGATGATTCATTGAAGCCTATCCCTTGCCCATCTAAGAAACTGTTGGACCTTGGATTCAAGTTCAAATACAACTCTGATGAGTGTGATGCAGGGGATTTGTGTGCTGAAGCTATTGAAGCATGCAAAGAAAAGGGGCAGATGCCATCTCTATAGAGACTGCAGACCGAGACATTT

>CsDFRb2

TCTGATTATGATCCATAGAGAATTCTAAAATCAATTCATCATCATTTATATTCTCTCAAAGACATGGAAGAAGAAGAAGGAGAGCATGGTGGAAGTGGAAGAACAGTGTGCGTGACAGGGGCTTCAGGGTTCATAGGCTCATGGCTTGTGATGACTCTTCTTCAACGTGGTTATCATGTTAGAGCCACTGTTCGCGATCCTGATAACAAGAGCAAGGTAAGTCATCTTCTAGACTTGCCGAAAGCAGGAACACACCTAACCCTGTGGAAAGCAGATCTAATGGAGGAGGGAAGTTTTGATGATCCAATTCATGGTTGTGATGGTGTCTTCCATGTTGCCACCCCTACGGAATTTAGTTCCAAGGATCCTGAGAATGAAGTAATTAAACCAACAGTGAATGGGGTTTTGAACATCATGAGATCATGCTCCAAAGCCAAAACTGTGAAGAGGCTTATCCACACCTCAACCACTGGAACTGTTGTCATCCAACCACAACCCCAACCCCAACCTGAACCTGATCAGTATGATGAGAGTTTTTGGACCGACATCAATTTCTGCAAGGCTCAAAAGATGACTGGATGGATGTATTTTGTGGGGAAAACAATGGCCGAGAAAGCTGCTTGGGAATTCGCTGAAGAGAATGGGCTTGATCTCATCACCATTCAACCCTCTATTGTAATAGGGCCTTTCATCACGCCTTCAAAGCCTCTCAGCATTGAACTGTCAATTGCCTTAATCACTAGAAATGAACGACTTTATCCAATGCTAACTCGAGCACGGGCCGTTCACGTGGTTGATGTATGCAATGCTCACATATACCTGTTGGAGCATCCGCAAGCCAAAGGAAGATACATCTGCTCGTCTCACTGTTTTACCATATTTGATCTCTCAAAATCGCTCAGTCAAAAATACCCAGAATTCAACATACCAACCAAGTTTGAGAGTGTTGATGAATCGTTGAAACCTATCCCTGCCTCGTCGAAGAAACTGTTGGACCTAGGATTCAAATTTAAGTACAACTCGGATGAGTACGATGTGGGAGACTTGTGCAGTGAAGCCATTGAATCATGTAGAGAAAAGGGGATGTTGCAACTATAGGGAGCAAGAAATTAAAAAGCCAACATTAGGATTTAAAATTTATAATACGCAAATCTATGTTTGATATGGT

>CsDFRb3

GGCATTCAAGTCATCATCTATTCTCTGTGTTTGGTTATCTATCAATAGAGAATATTAAAGTCAGTCATCAATTGTCCTCTCAAAGACATGGAAGGAGGAGAGCATGGTGGAAGTGGAAGAACAGTGTGTGTGACAGGGGCTTCAGGGTTCATAGCATCATGGCTTGTGATGAGACTTCTTCAACTCGGCTATCATGTTCGAGCCACTGTTCGCGATCCTGATAACAAAAGCAAGGTGAGTTATCTTCTAGACTTGCCGAAAGCTGGAACACACCTAACCCTGTGGAAGGCAGATCTATTGGAGGAGGGAAGTTTTGATGATGCAATTCAAGGATGTGATGGAGTCTTCCACGTTGCCGCCTATGTGCCTGTTCTTCCTGTCAACGATCCTGAGAATGAACTAATTAAACCAACAGTAAATGGGGTTTTGAACATCATGAGGTCATGCTCCAAGGCCAAAACTGTCAAGAGGCTCATCTGCACCTCAACCACCGGAGCTGTTACCGTCCAACCACCACCCGAACCTGATCAGTATGATGAGAGTTTTTGGACCGATGTTGACTTCTGCAAGGAAAAAAAGATACTTGGATGGCCATATTTTGTGGCGAAAACAATGGCAGAGAAAGCTGCATGGAAATTCGCTGAAGAGAATGGAATTGATCTCGTCGCCATTCATCCATCTCTGGTGATAGGGCCTTTTATCACGCCTTTGAAACCTGCCAGCATCGATATAACACTTGCCTTAATCACTAGAAATGAAGCTCTTTACCCACTGCTAAGTAGAGGATGGGCGGTGCACTTGGATGATGTTTGTAGTGCTCACATATATCTGTTGGAGAATCCTCAAGCCAAAGGAAGATACCTGTGCTCCTCTCACTGTTTTACCATTTTTGAACTCTCCAAATCACTCAGCCAAAAATACCCAGATTGCAACATACCAACAAAGTTTGAGGGCATTGATGAATCTTTGAAATCTATCCCAGCCTCATCTAAGAAACTGTTGCACCTAGGATTCAAATTTAAGTACAACCCGGACGAGTATGATGCAGGGGACTTGTGCAGTGAAGCCATTGAATTTTGTAGAGAAAAGGGGTGGTTGACACTATAGAGAGCAAGAAATGAAAAGGTCAACAACAATGAAGCTGGTTGGGACTCAAAATTTAGAATATGGAGAGCTATGTTTG

>CsDFRc

TTCACAGAGAAACAGGAAAAATGGAAGATACAAAAAGTAAGCTGCAGGAGGGAAGTGCAGTGGAAAGCCTGACCACCACCACCTACTGTGTCACGGGAGCTACAGGATACATAGGGTCATGGCTGGTCAAATCTCTCCTTCAAAGGGGCTACAGGGTTCACGCCACGGTCCGTAATCAAGCAAAGACATTGCATCTGTTACCATTATGGGGTGGCGGTGATCGGTTGAGAGTGTTCAGAGCTGATCTGCACAAAGAAGGAAGCTTCAATGAGGCTGTACAAGGCTGCAGCGGTGTATTTCATGTCGCTGCTTCAATGGAGTTCGAGGTTCCAGCAAAAGAGGACATTGACAGTTATGTCCGCGCAAATGTCATTGAGCCTGCAATCAATGGTACCTTAAACCTTCTCAAATCGTGCTTGAAAACACCATCTGTGAGGAGGGTTGTGTTCACATCATCCATCAGTACCATGACTGCCAAAGACAGTGCCGGAAAATGGAGAAGTGTTGTCGATGAATCTTGTCAGAATCCCATTGATCGTGTCTGGAAAACAAAAACAGGTGCCTGGGTTTATGTACTGTCAAAGCTTCTGACTGAGGAAGCAGCATTCCAATTTGCTAATGAGAACAGCATTGATCTTGTGTCAGTCATAACCACAACTGTTGCCGGTCCATTCCTCACTTCAACAGTTCCAACAAGCGTTCAAGTTCTCTTGTCGCCAATAACAGGCGACTCTAAATTCTCTCGAATCTTATCTGCTGTGAATTCAAGAATGGGGTCAATCGCTTTAGTTCATATCGAAGATATATGCAGTGCCCACATATTCCTCATGGAACATGATGGAGCAGAAGGTCGATACATATGCTCTGCCCACAGTTCTGTAATATCTCAACTGGTTGATCATCTTGCTAAAGAGCACCCTTTCTCAAATATGCAGAGGCTTGTAGAGGAAGAACATGGCTCAGTCCCCTCCGAGATTTCTTCAAAGAAGTTAAGAGATTTGGGTTTTAATTTCAAGTTCGGCTTGGAAGATATTATACACCATACTGTTAGTTCTTGTGTAGATTGTGGCTTTTTGTGTCCTACTGTAAACTAGATTGC

>CsDFRd

CAACATCACTGGTTTGACTTTGATTGCCAGAGAGAGAGAGAGAGAGAGAGAGATAGATCAGGACCAGGATCAGTATGATATGAGGGTGTTGGTGACCGGTGCTTCCGGTTACTTGGGCGGAAGGCTCTGCCACGCCCTCGTCAGGCAAGGCTATTCCGTACGGGCCTTCGTCAGGTCAAGCAGCGATCTGAGCTGCCTACCTCCGGCCGGCGAGTACGAGGGAGCTTTGGAGCTCGCTTACGGGGATGTGACTGACTTCCGGTCCCTCCTGGCAGCCTGCTCCGGCTGCCACGTCATCATCCACTCCGCCGCCATCGTCGAGCCCTGGCTTCCTGACCCTTCTAGATTCTCCACGGTTAATGTTGGAGGTTTGAAGAATGTATTGCAAGCATACAAGGAGAGTGAGACGACCATACAGAAGATAATATACACATCCTCGTTTTTTGCCGTGGGATCAACCGACGGTTATGTTGCAGACGAGACTCAGATTCATTCTGCCAAGTCATTCTGTACAGAGTATGAGAAATCAAAGGCAGTTGCAGATAAGATCGCCTTAGATGCCGCCTCGGAGGGGGTTCCAGTCGTGGTTGTCTATCCCGGAGTTGTATACGGTCCCGGCAAACTCACCGCTGGAAATATAGTCGCACGTTTGATTATTGAACGTTTTAATGGGCGGTTACCTGGTTATATAGGCTATGGAAATGATAAGTTTTCTTTTAGTCATGTTGATGATGTAGTAGAAGGGCACCTTGCAGCTTTGAACAAAGGTCGACCAGGCGAAAGATATCTTTTAACGGGTGACAATGCATCGTTCATGCGTGTTTTTGATATAGCTGCAGCCATCACTGAAACGAAAAGGCCTTGGTTTAACATTCCATTATCTGTCATTGAGGTTTATGGATGGATATCACTTTTTTTCTCTAAAATAACAGGCAAGCTTCCCCTGATCAGCCCCCCGACAGTGTATGTTCTTGGACATCAGTGGGCTTACTCTTGTGAGAAGGCCAAGGCAGAGTTGGGGTATAACCCTAGAAGCTTGCAAGAAGGTCTAGCAGAGGTACTACCCTGGTTGAAGAGCTTGGGCTTGATAAAATACTAGATTGATGATATTTGGCTTGTGATGTGGGTGTACATGTACTAATATTGCTGTATATTAGCAGCCTATCATGATATGAGT

2. Amino acid sequences of putative CsDFRs

>CsDFRa_long_38.70kDa

MKDSVASATASAPGTVCVTGAAGFIGSWLVMRLLERGYIVRATVRDPANLKKVKHLLDLPKADTNLTLWKADLNEEGSFDEAIEGCSGVFHVATPMDFESKDPENEVIKPTINGVLSIIRSCTKAKTVKRLVFTSSAGTVNVQEHQQPVFDENNWSDLDFINKKKMTGWMYFVSKTLAEKAAWEAAKENNIDFISIIPTLVVGPFIMPTFPPSLITALSPITRNEGHYSIIKQGQFVHLDDLCESHIFLYERPQAEGRYICSSHDATIHDLAKLMREKWPEYNVPTEFKGIDKDLPVVSFSSKKLIGMGFEFKYSLEDMFRGAIDTCREKGLLPHSFAENPVNGNKV

>CsDFRa_short_37.97kDa

MKDSVASATASAPGTVCVTGAAGFIGSWLVMRLLERGYIVRATVRDPANLKKVKHLLDLPKADTNLTLWKADLNEEGSFDEAIEGCSGVFHVATPMDFESKDPENEVIKPTINGVLSIIRSCTKAKTVKRLVFTSSAGTVNVQEHQQPVFDENNWSDLDFINKKKMTGWMYFVSKTLAEKAAWEAAKENNIDFISIIPTLVVGPFIMPTFPPSLITALSPITRNEGHYSIIKQGQFVHLDDLCESHIFLYEHPEAEGRYICSSHDATIHDLAKLMREKWPEYNVPTEFKGIDKDLPVVSFSSKKLIGMGFEFKYSLEDMFRGAIDTCREKGLLPHSFAEN

>CsDFRb1_38.5kDa

MEVVEVKNGYGGGGGTTVCVTGASGFIGSWLVMRLLQRGYYVRATVRDPDNTDKVKHLLDLPNATTHLSLWKADLDEDGSFDDAIQGCHGVFHVATPMNFVFVMDPENEYIKPTVDGVLNVMRSCCKAKTVKRIIYTSTMATIEYQQKPPSQYDESIWTDVDFCRARKMFAWMYLVAKTEAEKAAWKFAEENGPDLITVHPCSVIGPFITPYKPPCTSMALALITKNKAFYPMLTQGHAVHVDDVCNAHIYLFEHPQAKGRYICSSHSFTIFDLANSLSKKYPEYNIQTKFEDIDDSLKPIPCPSKKLLDLGFKFKYNSDECDAGDLCAEAIEACKEKGQMPSL

>CsDFRb2_38.47kDa

MEEEEGEHGGSGRTVCVTGASGFIGSWLVMTLLQRGYHVRATVRDPDNKSKVSHLLDLPKAGTHLTLWKADLMEEGSFDDPIHGCDGVFHVATPTEFSSKDPENEVIKPTVNGVLNIMRSCSKAKTVKRLIHTSTTGTVVIQPQPQPQPEPDQYDESFWTDINFCKAQKMTGWMYFVGKTMAEKAAWEFAEENGLDLITIQPSIVIGPFITPSKPLSIELSIALITRNERLYPMLTRARAVHVVDVCNAHIYLLEHPQAKGRYICSSHCFTIFDLSKSLSQKYPEFNIPTKFESVDESLKPIPASSKKLLDLGFKFKYNSDEYDVGDLCSEAIESCREKGMLQL

>CsDFRb3_37.54kDa

MEGGEHGGSGRTVCVTGASGFIASWLVMRLLQLGYHVRATVRDPDNKSKVSYLLDLPKAGTHLTLWKADLLEEGSFDDAIQGCDGVFHVAAYVPVLPVNDPENELIKPTVNGVLNIMRSCSKAKTVKRLICTSTTGAVTVQPPPEPDQYDESFWTDVDFCKEKKILGWPYFVAKTMAEKAAWKFAEENGIDLVAIHPSLVIGPFITPLKPASIDITLALITRNEALYPLLSRGWAVHLDDVCSAHIYLLENPQAKGRYLCSSHCFTIFELSKSLSQKYPDCNIPTKFEGIDESLKSIPASSKKLLHLGFKFKYNPDEYDAGDLCSEAIEFCREKGWLTL

>CsDFRc_39.09kDa

MEDTKSKLQEGSAVESLTTTTYCVTGATGYIGSWLVKSLLQRGYRVHATVRNQAKTLHLLPLWGGGDRLRVFRADLHKEGSFNEAVQGCSGVFHVAASMEFEVPAKEDIDSYVRANVIEPAINGTLNLLKSCLKTPSVRRVVFTSSISTMTAKDSAGKWRSVVDESCQNPIDRVWKTKTGAWVYVLSKLLTEEAAFQFANENSIDLVSVITTTVAGPFLTSTVPTSVQVLLSPITGDSKFSRILSAVNSRMGSIALVHIEDICSAHIFLMEHDGAEGRYICSAHSSVISQLVDHLAKEHPFSNMQRLVEEEHGSVPSEISSKKLRDLGFNFKFGLEDIIHHTVSSCVDCGFLCPTVN

>CsDFRd_36.85kDa

MRVLVTGASGYLGGRLCHALVRQGYSVRAFVRSSSDLSCLPPAGEYEGALELAYGDVTDFRSLLAACSGCHVIIHSAAIVEPWLPDPSRFSTVNVGGLKNVLQAYKESETTIQKIIYTSSFFAVGSTDGYVADETQIHSAKSFCTEYEKSKAVADKIALDAASEGVPVVVVYPGVVYGPGKLTAGNIVARLIIERFNGRLPGYIGYGNDKFSFSHVDDVVEGHLAALNKGRPGERYLLTGDNASFMRVFDIAAAITETKRPWFNIPLSVIEVYGWISLFFSKITGKLPLISPPTVYVLGHQWAYSCEKAKAELGYNPRSLQEGLAEVLPWLKSLGLIKY

3. Promoter sequences of putative *CsDFRs* extracted from SCZ genome data

>CsDFRa_promoter

AGTTTTGGAGTATCATGTGAATATATTCTTTCACTTATCACTATGTATTATATTTATTAATATTTTTCAATCACCTAATATAAATAATTAATATTTCACTTTTTTCACCAATCATGTTATCATAGAATATTATGTAATCGTACTTGTAGATCAAGAAGAGCACAAAATAAAGAGAAGTTATTGATTAATCCATGAGCAATATCACGTGACACTTTACATGTATTGTAGTTAGTAGAGAAAATAAAATTTACTATTTTACGTTAGGTGATTAAAGAATATAAAGAGGCATAAAAATTTATACTTATCTTGAGATATAGTCACATGATATTTCAATACTCATCAAAAATCCATCCAAATACCAAATTTTAATAATGTCTACATCACATCATTTTTTGGAAGGTTATTTATATAAATTCTCCAAAAGTAGCGTTATTCGTGACCAAAACGCTTTTCAAACGGGGGCGCACGCATATATATATATAAATGGTAGAGACCCTCAGCATTTTGGCACCTCCAGCATCCCCCAGTGCCACATGGGGGCTGCTAATGGCCAAAAAAAAAAAAAAAAAAAAAACAATTTTTTCAACCAATCACAATGGACCCCAGCACCCCCACTGGGGGTCCCAAGCTTTATTCATATATATATATATGAAAAATTATTTAATAATTTCGAAATATCACGTGATTATATTTCAAGTCAATTATCAATTACTTTAACTTTATGTTATTTTTCTATCCCCAACATAAATTATGGGTCTCTACTCCTCTCTATCAATCAAAATCTTATGGAGCACTACGTGTTCATACCCGAAGATACTGAACAATCACCTTATATATAGGATTTTTTTATTTTCTTTCCTTCTTATGGTTCATTTGTTTTTTTAATATGATCTACACTTTTTATCTTCTAAATTTCACAATAAAAATTATTTATACAAAAAATAATAATAATTTCATTAAATATCAATAAATAAATAAATAAATAATATTTTATTTTATAAAATAAACTATTTAAGTTCAAATTCATAGTTATTATAAATTTAAAAAGAAAAAAAATATAATCTATTTTTCTCATTTTAGAAAATAGACGATTTAAATTTGAATTCACAGTTATCATTCATTTTAATCATATATATATATATATAATCTGTGATTCAATGGAACTGATTTTTTTACACCAATCATGTTTATTACTAAATTTACGAAATAAACTATTAAGATGATCAATGTGGAGAAAAATTATATAATAGTTTTGGAGTATCACATGGATATGTTCTTTCATTTATCACTATGTATTATATTTATTAATATTCTTCAACCACCTAATATAAATAATTAATATTTCACTTTTTTTCACCAATCATACTATCATAGAATATCATGTAATCGTACATGTAGATCAAGAGAAGCACAAAATAAAGAGAAATTATTGATTAGTCCAGGAGCAATATCACGTGACACTTCACATGTATTGTCGTTAGTGGAGGAAATAAGATTTACTATTTTGCGTTAGGAGATTGAAAAATATGAAGAGACATAAAAATTTAGAATTATCCTGAGATATAATTCCGTGATATCTCAATACTCATCAATAATTTTTTGGAAATAAAATATATAGAGAAGGGTCCACATGGATAAAGCTCATATGTGGGGCCCAGAAATCACATCATAATGTGGTGAAAGTGGACGTAGAAGTATAAGTAGAGGTGGGCAGCTCCTAGTATTCTTCCATCGAGTCTGGTTTTGGGGTTTAGTTTAAGTGGAATGGTAAGTGGAGCACGTGCTTTCCTAGCCACAACAACGTCAAAATGACTGATACAGAGCGCCCTTTCTTTACACTTTATTTTATATATACACAACTATAATTTGCAAATTCCCTCAATATAATCCCAATCGCAACCATATCAAAATCTTCACTAATACCAATCCTACCACTTGTGTTTTACTTGTCCTCTCTTAAAATTTTTGTACTCCCAATTTTTTCACATTTTAATCCCATC

>CsDFRb1_promoter_1674bp

TGGGTGGTGGTGGCGGGTGGTGCGTGGTGGTGGCAGGTGGGTGACGGGTGGTGGTGGGTGGTGACGGGTGGGTGGTGGGTGATGATAGTGGTTGTGGTGGTGGTGGCAGGTGGGTAGCGAGTGAGTGGTGGGTGATGGTAGCGGTGGTGGTGGGTGGTGGTGGTAGTGGCAGGTGGGTGGGTGATGGTGGGTGGTGGCGGGTAGGTGGCGGGTGGTGGTGGTGGTGGATAAGTGGGTGACGGTGGTGGGTGGTAGGTGGTAGCGAGTGGGTGTGGTGAGTGGTGGTGGCGGGTAGTGGGGGGGGGTGGGTGGTAGTGGCGGAAGACGGTGGTGGAAAATGGTGACGGGTGGTGGCTAGTATTGGGTAGTAAAAAAAAAAACTGAAAACTCAAAATGGAAACATATTTTTTCTGTTTTTATTTTTTGGCCAAATTTATAAACTGAGACCACTGATTTATAAATCAAAAATTTTACCAAACAAATTTTTATTTAAATTTTTAAAATTTTACTAAATATAAATAAAAAATTAAAAATATAAGTATTACCAAACGGCACCTTTATTTTTTGGGCACATCACATAACTCCTTTAAAAAAAAAATCAGAAAAGAATAAAATAAAACAAAACAAATTAACACAGCCAAATAAAAAAAAAAGAATTCGACGAATTTGTAGCCTCTGGCACTGCCGATAATAAAAATACCCTATAACATGTATCATATCCGCATGTTCCGCGTGTTTGAGTCCGTTGCCACTGGCCACGCGAATCAGGATCGTCCCCTTAATTTTTTATTTATTAGGTTATTGGGCTTACTTTTAAGGGGATGCACCAATAAATTCTAAATTAATAACTTATACATTATTTAATCTTAAATTTTTTTTTATAGACTGAAAGATCCCATTATAATGTTTCAAACATCAGATTAATGTTTCGAACATTAAATTTCGAACATCAGATTAATGTTTCGAACATTAGATTGATGTGCAAATGAGACTAAATAGAGCATAAGAAAACTTTTAGGACTAAATGGTTCAACTTTTCAGAAACTAGGACTTATGGGTAAATTTTCTTACTTTTAAATCTAATTTTAATAATTGGAATAGTGTATATTTTAGAGTCATGATAGAGATCCCCTCTAATCACCACCCTACAACCCCACAGACACGTGGCGGGTGGTGATTGGAGGAGCAGGAAAAAAAGAAAAAGAAAATATAACCAATGAGAGAGAGGGGGTCAAAGGGGGTGGGGGAAAAGGAGACATGAAACATTTTTGTATATTTTAAAATTTATTAGATATATCATACTTATAAAAAATCAAGTTAATCAGATATCAGAAAGCATATGTTCGAAAAACTGTTTATTAATTTTTTTAAAAAAATAATTTTTAGTTTGATGATCCGAACACGTAATTTCTGATATCTAATCAACTTAATTTTTTATAAATATAATATACTCAACAAATCTTAAAATGTGAACGATTCATATCATCAAAATTAGGTCCAAAATTAAGTTCAATAACCCAACAAGTAAGAAATTAAGAAAAAAAGTGAAGGAGAGGATCTTGATTCCTGGCCACGCTTGTGGAGGCCTTGTCCTCGTGGCTCTGCAACCATGAATAAAAGCAAACTGATGACCAGTGAATACCCCTAAACTTCTCAACTTCTCTCTCTCTCGCA

>CsDFRb2_promoter

AGACTCTTCAAAACTTTTGTTGTTCCTCACAGACTTCACTGGGGCTGGGTTTGGACTTCTCTTGGCTCACCTACAAACACATGAATGAAACACATTTTTTGTGTTTTCATTTTTTGGCCAAATTTATAAACTGAGACCACTGATTTATAAATCAAAAATTTTACCAAACAAGTTTTTATTTGAGTTTTTAAAATTTTTGTAAATATAAACAGAAAACTGAAAACGTAAGTGTTACCAAACGGCACCTAAGTGTCCGTTCTTCTTAATCAATTATCATTCATGGATCGTTTGGTAATGACCTTTTTAATTGACTTTTTGACTTTTTTGATTTTTTTAGCCTGACACTTTTTTGTTTGTTTGGTTTTCTTGTTAGGGTTTTTTGGTTTTTTGACTTTGAAATGGCAAACTTTTTTTTGTCCTTTTGTATTTTTGACTTTTTAACTTTTTTGACTTTTTTTTTTTTTTTTTTTTTTTTGTCCTTTTATGATTTTTTAGTTTAGAAGGAAAATCAAATTGGGGCTCAATATGTCTTCTCATATTTCCTAATCACCTAATACAATACTCCACCTACCCATATTCTCCACCAATCACAATGTTATGGAATACCACGTGTCTATATTCTGGAATTATTGAACAATTACACCTTCAGGATGAGGTGAATTATTAGCAAGGGAAACAACTTTAAGGGAAATTATTAAAGGCTGGTTTGTCATAGTCTAGAAAAGTTAAAAAGTTATTAAAAAAGTTAAAAAGTTATCAGTATTCACAAAAAAATATATATATAAAAAAAATAAATTTACACAAAAAAAATACAACCCAAAAAGTCATTGACATGATGGTATAGTCCAAACCAGATGATACAAATTACCTAATTGGCTACACAAAAAATTTACAAAAAAACGGTGTTGTTTGGCCCATGGGGGCCTACCATGTGTCCCGTTTTGGTAATCGAAACTGTTCAATATATAGAACTCGTCGAGTACTATATACACGTGAAAAATCACGTTGATCGGATATCGATAGCTATGTGATCAAATCAAATTTTTTTCAGTCGTTTATTTTTAATCGTCTATTTTTTTATACAAAACTGCTTTGATCACATAACTATCGATATCCGATCAACGTGGTTTTTTACAAGCATATAGTACTTAACGATTTCTACATATTGAACAGTTCGGATGATTGAAATGAGACCCGTGGTAAGCCCCTCTAGTCCGATGAAGGATTAAAAGAAAGCCTCATCTTATATATATAAATCTGAAGCATGTCTGCCCCTATACACAATTCAAAGCATTCCGTATTGCTTCCTTCAATAGTCACTGTCACTATGCATAGGGCATAGGAATAAAATGGTAATTTTGTTATTTTATAAAATATTTTAATACACATTTTTTCATAAAAAGTCAAAAAATTATCCCAAATAACCATCTTTTTTCCCTCAACTTTTTTGTTCAAAAGGCCAAAAAGTGCATTTATTTAGGACATTCCAAACAACCCTAAATATTTCTGGAGTACCACGTGTCTGTACTTTTCAATCAATCATCATTTAATGTGTCATTCATGTTCTCCAATCACCCAATATAATACCTTACCTATCCACATTTTCCATCAATCATAACACTATGGAATACCACGTGTTCATATTCTGGAATTACTGAATAATTACCAACTTTTGGCCTTTGTTTGACATAAGCTAAAATGAGACTTTTTAACTAAAATAGCCACAAAAAGACAAAATAAACTTTTTAACTTAATAAGACAAAATGTTACATTCAAACTATAATCATTTCATTTTTTCTCTCACAAAAGTCAAAAAAACTAAAAAACACTTTTTAACCCAAACTAAAATAATTAAAAAAACTAAAAAAACCAACCCAAATCGCAAGCAAACCTTTGTCCGTCTCATAATTGGGGGTAGCATCTATTCTCTCTCTCTCTCTGATTATGATCCATAGAGAATTCTAAAATCAATTCATCATCATTTATATTCTCTCAAAGAC

>CsDFRb3_promoter

TGCTCCTCAAAAGTTTATTAGTCTAAATAATAGAACTCATAATCTATAGATTAGTATTTTAAAAGATAAAAGACAATATAAAAGAATGAAAATTGTTTTCAGTAAAAAGGATTGGATGATCCTGTTCGGAATTATGGGTCCATTTTAATGATTGAATTCGTTCATTTTTTAAATCTACCTAGAGGACGACTAGTTAAATATTTGGAAAAACTTTGAAAGGATGAATCAATATAATTTGAAAATTATTTATCGTTAATACAATTATAATATCTATGTTGAGTTATAAAAGATAGCAGGTGTGAAATAATATATCTAAAATTGTGATCGACACTCTTTGAAGACATTAAAAGTATACATATGATTAAATCAATATTAAAATTTCAAACAATATCTTTCTTAATAAATGTAATCAATTAATTTGTTGACTTTTCTATCTTCTTCTCTTATATGTAGATTAGGGCTTGTAATTGATAATAGTTATGAGAAATCAATTAATCAATTTCTTTTGTTGGAATTTTTTAATTTCATTATTTGATTCTTATGTTTCATGAATATATAGGAGAGTGACTTGCTTAATTTTATTTTATTTTTTAATTTATAGAGGACTCATTAAACTTTAAATATTAAATAAATTAGGAGGAGCAAAATTATATAATTTGAAGATAATTAATAACTAATACATGCTATTTTCATGAAATTATTATAGTCCCCTTGGGGTCAATTCTTCTACATCATCAACTTTGATTAATATCTATTAAATGGTGGTTTCTTTTGTTGGAATTTTTTAATTTCATTATTTGATTCTTATGTTTCATGAATATATAGGAGAGCGACTTGCTTAGTTTTATTTTATTTTTTAATTTATAGAGGACTCGTTAAACTTTAAATATTAAATGAATTAGGAGGAGCAAAATTATATAATTTGAAGATAATTAATAACTAATACATGCTATTTTCATGAAATTATTATAGGGGACCATAGTCTCCTTGGGGTCAATTCTTCTACATCATCAACTTTGATTAACATCTATTAAATGGTGGTTTCTTTTGTTGGAATTTTTTAATTTCATTATTTGATTCTTATGTTTCATGAATATATAGGAGAGCGACTTGCATAATTTTATTTTATTTTTTAATTTATAGAGGACTCGTTAAACTTTAAACATTAAATAAATTAGGAGGAGCAAAATTATATAATTTGAAGATAATTAATAACTAATACATGCTATTTTCATGAAATTATTATAGGGGACCATAGTCTCCTTGGGGTCAATTCTTCTACATCATCAACTTTGATTAACATCTATTAAATGGTGGTTTCTTTTGTTGGAATTTTTTAATTTAGTTATTTGATTCTTATGTTTCGTGAATATATAGGAGAGCGACTTGCTTAATTTTATTTTATTGTTTAATTTATAGAGGACTCGTTAAACTTTAAATATTAAATAAATTAGGAGGAGCAAAATTATATAATTTGAAGATAATTAATAACTAATGCATGCTATTTTCATGAAATTATTATAGGCCCTTGGGGCCAATTCTTCTACATCATCAACTTTGATTAACATCTATTAAATTTTGGTTTCTTTTGTTGGAATTTTTTAATTTCATTATTTGATTCTTATGTTTCATGAATATATAGGAGAGTGGCTTGCTTAATTTTATTTTATTTTTTAATTTATAGAGGACTCGTTAAACTTTAAATATTAAATAAATTAGGAGGAGCAAAATTATATAATTTGAAGATAATTAATAACTAATGCATGCTGTTTTCATGAAATTATTATAGGGACCAGAGTCCCCTCGGGATCAATTCTTCTGCATCAACTTTGATTAACATCTATTAAATGGTGGTCGACTTTTCCCATTTAATAATGGAAACATCATTATATATGTAGTCTCTCCTGTATTGAGGCATTCAAGTCATCATCTATTCTCTGTGTTTGGTTATCTATCAATAGGGAATATTAAAGTCAGTCATCAATTGTCCTCTCAAAGAC

>CsDFRc_cp1_promoter

TGGGATCCGGGATCTAGAATTCGGGATCTGCTCACTTCGGCTGAGCTCTTGTCTCCTCGGACCTAATCATCTAGGCCGAGATCTTGTCTTCTAGGATCTAGGATCTGGGATCTGCTTACTTGGGCTGAGCTCTTGTCTCCTTGGACCTAATCATCTAAGTCGAGATCTTGTCTTCTGGGTACCAGTTTTGGTTGTTGGTGCCGACATGTCCTGTAATGGGGATGTTCGGTTGCATCACATTGTTCTGCATCATGAGGTTGACCACTGTCTGTAGATTTGTCAATATATTTGCCATGTTTTTCAGTTTCCTCGTCTTTTCTCCGGCATTTGTCCCTTCACACCTGAAGGTCCTTTTCATAGTAGTACTCATCTCAGCTACTCTTCTTGTTCTCTTCCTGGCCTTTAGCAACATTATTCGCCTCAGGATTCACTTCTTGCAATTTTTCGTTATGGTGCCTTCCTTCAATGATAGAATCTACATGCTCCTCCTCCTTACTGTGGATGTCGTCCCTAATCCTCACTTCCTGTTCATTGTTGGCCATCTATCCTTACTAAATTCCCACAGACGGCGCCAAATTGTGCGTGTTGATTTCTGACCACACAACCAAAAGGGGAATTCAGCTGGAGAGCACCGGCAGAATAGATTAAGCCCGAACAACCTATCAGAGAAAGACAAGAATCCGGCGGTGGTTCATCCACCCGGGGCCACTCTGACGCTCAAGTCAGTAATCAAATATCTCTTAAAAATAACTAATAGTGCTTAACTCACTAGACAGAATATAAAAAGCCTACCTTAGATCCATGGGGGTGCCTCACTTTTATAGTGGTCAGTGTCCAACTTCCCCTGGAAGTCTTACTTCAAGGGACTCTATCCTTTATAAGGATAACATCTATCACAACTCACACTATTCAGATTAAGATGAACTTTCTCAAGAGTCGCATCTCTATCCAAATAGGTAGTTTGATGTTATCTAGGACTCTAGTCCTCTATCCAAATATCACAGATTCTTGTATTATTGCACCATATCTCGGCATCATGGTCCGCCCATGTGGTACCTCGGCTAACAACCTCGGTCACATGGACTACCCAAAAATATGTCCCCACAGTTATAATCAATTTTCTTTTTTAATTATTATCTTATTTTACTTTGATTCATGAGAATTTTAGTTATAATGTCGATATCTTCGTTAGTTATAATCAATTTTTTTTTTATAGCTATTATCTTATTTTACTTTGATTCATGAAATTTTTAATTATATCTCATGTGTTATTTGATTATGATTTTTTTTTTTAATTATAAAATACACGAGGACAAAACCTTTGAAGGTACACTACAAAAAAATAAGGTTTTAGAGACCAAAATTTAGGGACAAAATTATTTTTTAGGGACCATATAATTTTACTGCGAAAAGTGTCATTATATTTTTAGCAACAAAAATTAGACATTAGTGATGAATTATTATCATTTTGAGACCAAATTATTTTGTTTCTAAAAGTCTATTTATCAGTGACATTTTCAAAATTTTGTTGTTAAATGCCTTGTTTTTGACTTTTCGGGACGACCTTTTAGCGACTATCTTCATGACGATGGAAATTAGTCCATAAATATATTTTGCAACAAAATTTTTACTTAGAGATCAAAACATTTAATTTTTAAAACTCTTAAATATTGTAATGGTATAGTCTTTGCCTCTAACCTATGAACTCCACCGACATTAACAGTTGCCTCAAGTCCCCATGCACATTAAAATAATGGATATAGATAAAAAGATTCCGCACTGGCCATATAAGGACTAGAAACAACCAAACATTAATTATTTCAAAAGATTAGTACAATATTAGTTAAAATGACTAAGCGTTGCCAACCTCTTTTTCATTGCCAATTAATATCTATTATCTATACCCATTCATACACTCATGTCTATTTATAATAGTATTCATATTCATTGACACACACAAATCTATCTGTTGAGACTTTTTTCACAGAGAAACAGGAAAA

>CsDFRc_cp2_promoter

ACCACCTACTGTGTAACGGGAGCTACAGGATACATAGGGTCATGGCTGGTCAAATCTCTCCTTCAAAGAGGCTACAGGGTTCACGCCACGGTCCGCAATCAAGGTCGATTTAACTTATCTCTCTCACTCTATTTCATATATTTTTAATTTTCAATTTTGATGACATAACAATATAAACATAATATGCGATAGAGATTACAAACGATCTATGTTCTTTTTTTTTTTTTTGAAGTGGAACTAATTGAATTGAAAAATAATTAGAAGTATGCAACATATAAGAGTTTTAGAGATCAAATATTTTGATTTAAAATATTTTGGTTTTAAAAGTGAAAATTTTGCTGTAAAAGACATTATTGTGAGGATAGTCGCTAAAAAGTCATCTCGAAAAATAAAAAATAAGGCTTTTTAGTAACAAAATTTTGAAAATGTCACTAATTAAATAGACTTTTAGGACAAAATAATAGTCTTAAAATGGTTTATTTTTGAGAAAATTAGTCTAAGGTCCCTCTCCCAATTTTAGTAATGGAAGAGATCTACAATATTATATTATGATGTTTATGGTCCCTCAACCCCACACCACATGTATTACAGAGAAATTTGGTATAAGTTTGAAACCTTCACCTCTCTCTCTCTCTCTCTTTTCTCTCTCAGATTTACGCCTCAACTCTTCTCTTTCCTCACTGCTCTGCTCTCTCTTTCGTCAATCTCACTCGATCTAGCTCGTCCTCTCTTCTGACCAGTTCGCTTTCCCCACTCTTTATCAGAAGATATCGGGATTGAAGTGTTTAAGTTCCCCTCTCTTTATCAGAAGATATCAGGATTGAAGTGTCCAGGGATAGAGTTACTTTGCTGAGCCTGCTGCAAATGCAATGTCAGTGCATAATTGTTCACCTGAAACCATATACCGCGAAAGCAGGAGAACTGTTAGCCCATAAATGTGTTTCCAAAAAAAAATTTGCGAACTAACTTAAGTTTGTAACAAACATGCAGGGCCATTGCCATGCTACAGATGCTCTTTACCACAACGAATACAAATACTGTTACATATAGAAAAGAGATTGTACATTATTATTATTTATTTATTCTTTTAACCACTTTTTTTTGTTTTGATAAATAAATTATTCTTTTAACGTCTCTGGAAAACATACTTACATGAATCATATCTTTTCACAGTTTGGATACAAGTCCAACTGAATACCATGTGATGATTGGAGTTTTGCTTACTTTTGGGTTTTCTGAACTCGTTTTTATACAGTTTAGCTATGTTTGCTTTCTCAGAAAATGAGTTTAATTTGATGTGGTCCAGCCCGCCCGGAATATTTTTAATTATATCTCATGTGTTATTTGATTATGATTTTTTTTTTTAATTATAACTCAAAAAATATAAAATTATAATTTTCTTTAATTATAAAATACATACGTACAAAACCTTTGAGGGTATAGGCCCTATTTGGTAGAGCTGTGGGAAAGTGCTTATAACTTATGTCTAAACAGCTTATAACTTATAACTATGAACATAAGCTGATTTCTGACTTATAAATTAAGCATCTCCTACCCCACTCAAAGAATAAGCTGATTTTTGACTTTCAAAAAACTATAAACTAAACCAAACACATCAAAATAACTTAAATTAAACTGAAAATTAAAATAAACAATTTTTAAACGGCACTAAACAGGGCCATACTATTTCCCTCTAACCTAAAAACTCCACCGACATTAACAGTTGTCTCAACTTCCCATGCACATTAAAATAATGGAAAAAGATAAAAAGATTCCGCACTGCCATATAAAAGGACTAAAAACAACCAAACATTATTATTTCAAAAGATTAGTACAATATTAATTAAAATGACTAAGCGTTGCCAACCTCTTTTTCATTGCCAATTAATATATATTATCTATACCCATTCATACACTCATGTCTATTTATAATAGTATTCATATTCATTGACACACACAAATCTATCTGTTGAGACTTTTTTCACAGAGAAACAGGAAAA

>CsDFRd_promoter

TGCAAGTACATTTCCTCCCTTTTGAGTGTTTCAGTAATCTCTTATTGAATAGCCTGTTGGGCTTGCTAATTATCCGTAGAGTGGGGACAGGCTTGTAATCTAGCAAGTTTAGCCTTCAAATCATTGAATCTATGAGTGTTATTCCCAAACTCTCGCATACTCCAACTTTTCAAGCCTACTTCACACGATTTGAGTCTTTGACACACAGAACATTGGAGAACCCAAGAAGTTCCTTGCCATTTGGAAGCTATAACTTCTTCACACATCGGGGAGATGGTACACATTGATTTAAACTTGAATAACCTAGGCACTCTTTTAGGGGGGATGACAGTATTGAGGAGAATACGACAATGATCCGATCCAATGAAAACTTCATGAAACACTTGAGCATAAGGGAACATATCTCTCCATTTGACTGTGGCAACAACTTTATCCAACCTTTCATGAATATGAACATCACCACCTTGGTTATTAGTCCATGTTTAAGCTTGCCCATTAAATTCTAAACCCATAAAAGCACACTCATTAAGGAGGTTCTAAAAATTCTCAATCCTCCTAGCATTAGGCTCAACCCCACATTTTTTCTCCCAAACAGACCCAATTTCATTAAAATCTCGTATGCAAATCCGAGGCAGATTATTCTCTCTAGCGATGGACCGAAGATAGTGCCAAAAAGCCACCCTTTGAGCCAGCCTAGAAAGGGCATAGATGGAGGTAATTAACCAATTTTGATCAGAGCCAGAGATCAAATAGCAAAAAAAGATTGCTCCCCTTATGTCTTACATCCAAACCAACTTCATGAATCCACCACAAAGCCAAGGCGCTTCCTAACTAGTTCCAATTCCGCTCGCTTATTTTTCGTCTCCGTCTTCTTGACGCCATCAAGAAGATTATTCCAAGACAATGGGTTTTACTCAACCCTCGCAAGATCTGGACTGTCTGGGGTCGCCCAATCCCCTGACAGCTCCAGCTTATTATAAACATGAACCTTGTGGCTGTTTGGGGCCAGCCACTAACGCCCTGTTCAAGTTATCCCCCTGAGAATCCAAAGGATAGGTATTGGGTACTTGGGAATAGAAATCTGTCTCCTCCACTGAAGCACTAAGATCATCACGAATGACAACATCAACTAGCTCTTGATGAGCATGCTTGCTTGACTTCTTGCCCTGTTTACTAGAGGAAGTACCCCCTCTATTTCTCCTGAAGTTACCCAATCCCCTAGCAGTAGTAGCACCATGAAAGGGGTGGATATGTAAGGGTGAGCTCATGGGCTAACTTAATAGCCTTGGATGAAGATTCAAGCTGTAGGTCCTCCTCAGGGGTCTTCCTTATCTTCCATTGAAAGCACAAGATAAGAATGCAGTGGATACATTTTTGCAAAGGCAAGCTGTTTGGATATGGAGATAGGGCTAGAAGAAGGGCTATCTTCATGAAAAGAAGATGGATAAGCTTTTTGCTTCTTTATCGTCTTTAGCGATAAGCACTAAGAATCAAGAAGTCAAGAATCTCTACTCTAATAGGCTCAAAGCTTTTTGAGAAATTGAGAAGCTTCAACTGGAGCTACTACTCATTCAAGCCCAAGAGAAGACACAAAGACGTTCAACCCTGATTCCCTCAAAAGCCCCTTTTCAATAAAAGATAGGCAGATGAACACCTTAAGCCCAGGCTTTCTAAACTTTACATATTTTTCTACATGGTATCAGAGCAGGTTTAGAACCTATTATTTTCGCTTCCCCGGCGGGCGGCAACCTCACCTTAGCCGCCCGCCGGACCTCAACCTATTCCGCTGTCATCTCTATCAATACATATAGCTTCCAGTTCGCCGCCACCAAAGGCGACTTCTCTACATACAAAGATCAACCATTCTCATCTCTCTCTGAGATGAAGAGGCCAACCTCTCTCTCTCGGCAACTCTCTCTTCGGTTTCTCTCTACACCACCTTCTCAACCTTTTAGTTCTACTACTGTGACCCTATCTATGATGTTTTTGGTGTCTCTGCA
